# Pediatric poverty and county-level cardiovascular mortality in the United States: A national cross-sectional analysis

**DOI:** 10.1016/j.ijcrp.2026.200722

**Published:** 2026-10-30

**Authors:** Kevin Babapour Digaleh, Mahdi Bouchekouk, Ronen Bina, Wyatt House, Toshihito Gomibuchi, Alyster Alcudia, Medha Tekriwal, Suyog Mokashi

**Affiliations:** a Lewis Katz School of Medicine at Temple University, Philadelphia, PA, 19140, USA; b Alice Walton School of Medicine, Bentonville, AR, 72712, USA; c Temple University Hospital, Division of Cardiovascular Surgery, Philadelphia, PA, 19140, USA; d Carnegie Mellon University, Pittsburgh, PA, 15213, USA

**Keywords:** Cardiovascular mortality, Socioeconomic factors, Pediatric poverty, Geographic disparities, Ecological analysis, Population health

## Abstract

**Background:**

Geographic disparities in U.S. cardiovascular mortality are large, but how readily available county socioeconomic indicators compare in their associations with it is unclear. We compared county poverty and unemployment measures as correlates of an AHRF-defined cardiovascular mortality composite.

**Methods:**

Cross-sectional ecological analysis of the 2024–2025 Area Health Resources File (AHRF). The outcome was the crude 2021–2023 rate of ischemic heart, cerebrovascular, and other cardiovascular deaths in counties with all three components observed. Measures were 2023 overall, pediatric (ages 0–17), and family childhood (ages 5–17) poverty and 2019–2023 overall, female, and White unemployment. Connecticut was excluded from county-level analyses. We estimated Spearman correlations with bootstrap 95% confidence intervals (CIs), paired comparisons, and partial rank correlations adjusted for age structure, rurality, racial and ethnic composition, and state.

**Results:**

Among 1982 counties, rates differed across Census divisions (p < 0.001), from 348.5 per 100,000 (East South Central) to 233.9 (Mountain). Unadjusted correlations were larger for poverty (ρ 0.524–0.612; pediatric poverty ρ = 0.612, 95% CI 0.581–0.641) than for unemployment (ρ 0.141–0.209). Pediatric poverty exceeded overall poverty (difference 0.088, 95% CI 0.071–0.107) but differed negligibly from family childhood poverty (0.009). After adjustment, partial correlations were 0.530 (95% CI 0.495–0.563) for pediatric poverty and 0.165–0.209 for unemployment; unemployment adjusted for poverty was near zero (0.029, 95% CI −0.020 to 0.077).

**Conclusion:**

Poverty measures, including pediatric poverty, showed larger associations with an AHRF-defined cardiovascular mortality composite than unemployment measures, before and after adjustment. These ecological associations with crude rates identify candidate county-level markers, not causal effects.

## Graphical abstract

**Figure:**
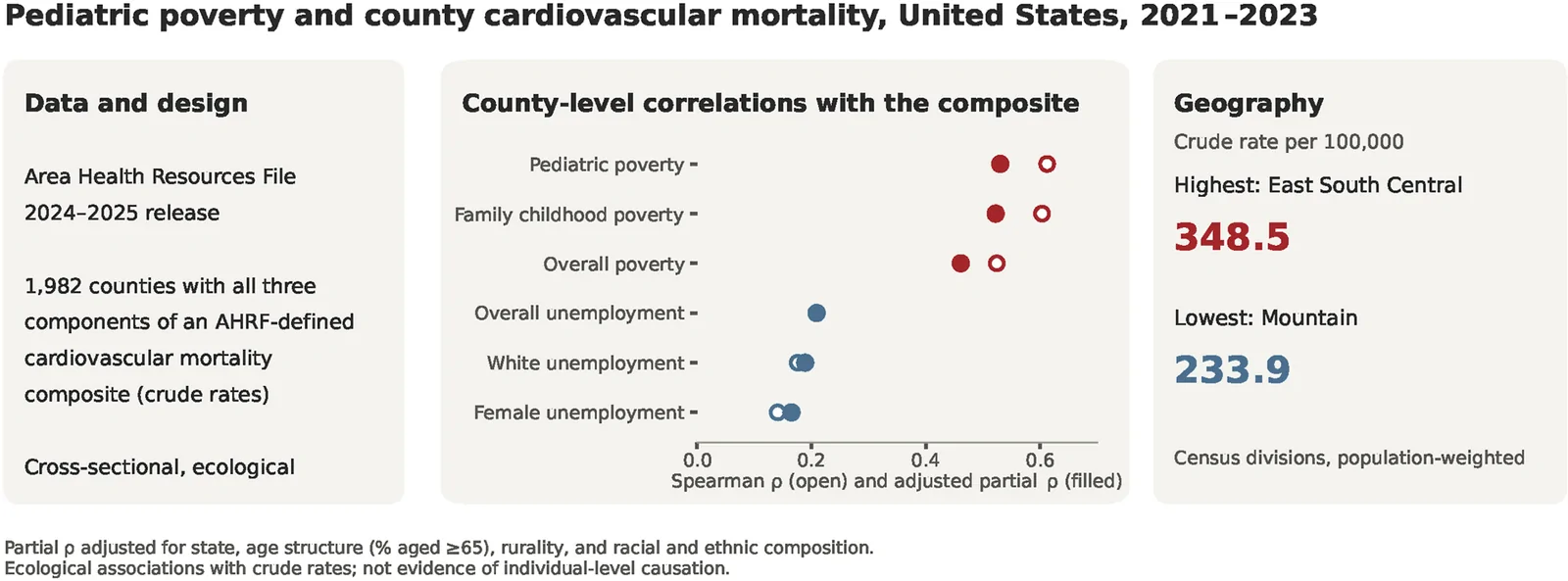

## 1 Introduction

Cardiovascular disease remains one of the leading causes of death in the United States, and substantial geographic variation in cardiovascular mortality persists across U.S. counties [1], [2]. Long-recognized high-mortality patterns extend across the South, Appalachia, and the broader Stroke Belt, with the highest county-level rates concentrated in a band from Oklahoma through Mississippi and into eastern Kentucky, and the lowest rates observed in portions of the Mountain West [2], [3]. These disparities have not narrowed uniformly over time; recent national analyses indicate that the gap between high- and low-mortality regions has remained large and, for some comparisons, has widened in the past two decades [4], [5]. Clarifying the county-level correlates of this persistent geographic variation remains a public health priority.

A growing body of county-level evidence indicates that socioeconomic conditions and area deprivation are consistently associated with cardiovascular mortality [6], [7], [8]. In recent county-level work, area-based deprivation indices have explained a substantial share of the variation in premature cardiovascular mortality, and changes in county economic prosperity have tracked changes in cardiovascular mortality among middle-aged adults over time [7], [9]. State-of-the-art reviews of the social determinants of cardiovascular health identify economic stability, including income and material resources, as an important social determinant of cardiovascular health [6], [10]. Poverty, in this context, can be understood as a measure of chronic material deprivation that may reflect upstream constraints on access to preventive care, food and housing security, educational and economic opportunity, and exposure to chronic psychosocial stress.

Individual-level literature links childhood adversity, including socioeconomic hardship among other adverse exposures, to later cardiometabolic risk through biological, behavioral, and social pathways [11], [12]. Work focused specifically on early-life socioeconomic conditions and adult cardiovascular disease has identified poor childhood socioeconomic conditions as a risk factor for adult cardiovascular disease, with proposed mechanisms including chronic stress and inflammation, accelerated trajectories of blood pressure and adiposity, and indirect effects on health behaviors and educational and economic attainment [13]. Longitudinal cohort studies and systematic reviews of childhood socioeconomic circumstances and adult cardiovascular outcomes are consistent with a life-course association in which childhood socioeconomic disadvantage is linked to later cardiovascular risk [14], [15]. This individual-level life-course evidence motivates examination of current pediatric poverty prevalence as a county-level signal of broader socioeconomic conditions, although ecological associations cannot establish individual-level causation. The relative associations of pediatric poverty, family childhood poverty, overall poverty, and unemployment measures with county-level cardiovascular mortality remain less well characterized, leaving open how readily available county-level indicators compare in their associations with cardiovascular mortality.

In this national, county-level analysis we therefore had two aims: first, to characterize contemporary geographic variation in an AHRF-defined cardiovascular mortality composite across U.S. counties, Census divisions, and states; and second, to compare the county-level associations between cardiovascular mortality and three poverty measures (overall, pediatric, and family childhood poverty) and three unemployment measures (overall, female, and White). Pediatric poverty was evaluated as a county-level correlate of cardiovascular mortality rather than as an individual-level causal exposure.

## 2 Methods

### 2.1 Data source

We performed a retrospective cross-sectional analysis using the 2024–2025 release of the Area Health Resources File (AHRF), a publicly available county-level dataset maintained by the U.S. Health Resources and Services Administration [16]. Within AHRF, cause-specific mortality counts originate from the National Center for Health Statistics (NCHS) Mortality Detail Data Files; population denominators from the U.S. Census Bureau Vintage 2023 County Population Estimates; poverty estimates from the 2023 Small Area Income and Poverty Estimates (SAIPE) program; and unemployment estimates from the American Community Survey (ACS) 2019–2023 5-year tables. Covariates for adjusted analyses came from the same release (Supplemental Table S2).

### 2.2 Study population and geographic units

The analytic universe comprised all counties and county-equivalents in the 50 U S. states and the District of Columbia (3153 AHRF county records); U.S. territories were excluded. Connecticut's AHRF representation reflects the state's 2022 administrative restructuring from 8 legacy counties (FIPS 09001–09015) to 9 Planning Regions (FIPS 09110–09190), and both row sets are present in the dataset. The file also carries a record for the former Valdez-Cordova Census Area, Alaska (FIPS 02261), dissolved in 2019; 2021–2023 deaths for this area are carried at the legacy code, whereas population and predictor data are carried for its successor areas (FIPS 02063 and 02066), so none of these three records could contribute to county-level rates.

### 2.3 Cardiovascular mortality outcome

The cardiovascular mortality outcome was an AHRF-defined composite constructed as the sum of three mutually non-overlapping NCHS-coded 3-year average annual death-count categories for the 2021–2023 window: ischemic heart disease (ICD-10 I20–I25), cerebrovascular disease (I60–I69), and other cardiovascular disease (I00–I09, I10–I13, I15, I26–I51, I70, and I71–I78), as defined in the AHRF User Guide. The primary outcome was the strict complete-component composite, defined only when all three components were observed for a county. A partial lower-bound composite, the sum of observed components when at least one was observed, was retained as a sensitivity outcome. AHRF suppresses 3-year average death counts for counties with fewer than 10 occurrences and carries suppressed values as missing, indistinguishable from data not reported for a geographic unit; the partial composite therefore counts no deaths for missing components and is a lower bound for counties with one or two missing components. Some missing values are structural rather than suppressed, as for the two Alaska successor areas described above, which have population but no mortality data. County crude rates per 100,000 were calculated using the 2023 Census County Population Estimate as the denominator. All rates are crude. Because HRSA does not formally label this sum as a unified construct, and because ICD-10 circulatory codes I80–I89 and I95–I99 are not included in these categories, we refer to the outcome as an “AHRF-defined cardiovascular mortality composite” rather than total cardiovascular mortality.

### 2.4 Socioeconomic predictors

Three poverty predictors were drawn from the 2023 SAIPE single-year estimates: percent of all persons in poverty, percent of persons aged 0–17 in poverty (pediatric poverty), and percent of related children aged 5–17 in families in poverty (family childhood poverty). Three unemployment predictors were derived from the ACS 2019–2023 5-year tables as percentages of the civilian labor force aged ≥16 years: an overall unemployment rate (Table B23001), a female unemployment rate (B23001), and a White unemployment rate (C23002A). The ACS White category includes both Hispanic and non-Hispanic White persons, and ACS unemployment estimates reflect a 5-year pooled window rather than a single calendar year.

### 2.5 Geographic aggregation

For state- and Census-division-level summaries, we computed population-weighted aggregated crude rates as the sum of 3-year average annual deaths across contributing counties divided by the sum of 2023 county population estimates across the same counties, multiplied by 100,000.

### 2.6 Statistical analysis

County-level associations between cardiovascular mortality rates and socioeconomic predictors were assessed using Spearman rank correlations, with two-sided p-values and pairwise complete-case sample sizes reported. Rank-based methods were used because county-level crude-rate distributions were non-normal and potentially influenced by outlying low-population counties. Differences in county-level rate distributions across the nine U.S. Census divisions were tested using the Kruskal-Wallis omnibus test. Statistical significance was defined as p < 0.05 (two-sided). The six Spearman correlations with the strict composite are reported as unadjusted associations; partial-composite and Kendall τ correlations were sensitivity analyses. Across these 18 tests, p-values were also adjusted with the Benjamini–Hochberg false discovery rate procedure. Analyses were performed in Python 3 using pandas, scipy, and statsmodels.

### 2.7 Confidence intervals, paired comparisons, and adjusted analyses

These analyses were added during peer review and selected after preliminary results were available (Supplemental Methods). Ninety-five percent CIs for each Spearman ρ were estimated by county-level bias-corrected and accelerated (BCa) bootstrap (10,000 resamples). Three paired contrasts (pediatric versus family childhood poverty, pediatric versus overall poverty, and overall poverty versus overall unemployment) were estimated on the same resamples and tested with centered bootstrap p-values and Holm's step-down adjustment. The main adjusted analysis estimated equal-weight county partial rank correlations: outcome and exposure ranks were each regressed on covariates and state fixed effects, and the residuals were correlated (Pearson). Model A included state fixed effects only; Model B (main) added the percentage aged ≥65 years, the 2023 Rural–Urban Continuum Code (nine categories), and the percentages of non-Hispanic Black, Hispanic, and American Indian/Alaska Native residents; Model C added the percentage younger than 65 years without health insurance, with changes from Model B interpreted as attenuation, not mediation. Each model included one socioeconomic measure, except a joint model of overall poverty and overall unemployment. Negative binomial models of composite deaths (log-population offset, Model B covariates, state fixed effects, state-clustered standard errors) estimated complementary, population-weighted rate ratios per 1-SD difference. Sensitivity analyses used Census-division fixed effects, three-category rurality, Spearman correlation of residuals, and contiguity-based spatial standard errors.

### 2.8 Sensitivity analyses

Two sensitivity analyses of the unadjusted associations were conducted. First, all county-level correlations and geographic summaries were re-estimated using the partial lower-bound composite, which retains counties with one or two missing NCHS components and uses a lower-bound numerator for them. Second, county-level correlations were re-estimated using Kendall τ as an alternative nonparametric metric.

### 2.9 Connecticut handling

Because Connecticut's 2022 transition from counties to Planning Regions affected the geographic units at which different AHRF data sources are reported, 2021–2023 NCHS mortality data are carried at the legacy county FIPS, whereas 2023 Census population, SAIPE poverty, and ACS unemployment estimates are carried at the Planning Region FIPS. Because no Connecticut row contained both a mortality numerator and the population and predictor data required to calculate matched county-level outcomes, Connecticut was excluded from all county-level analyses. For state- and Census-division-level population-weighted rates, Connecticut was included via a state-level aggregation in which the mortality numerator was summed across the 8 legacy counties and the population denominator across the 9 Planning Regions, both row sets fully covering the state.

## 3 Results

### 3.1 Analytic sample

Of the 3153 AHRF county records for the 50 U S. states and the District of Columbia, 17 were Connecticut records (8 legacy counties and 9 planning regions) and 1 was the dissolved Valdez-Cordova Census Area. Of the remaining 3135 county-equivalents, 1982 (63.2%) contributed to the strict primary AHRF-defined cardiovascular mortality composite, which was defined only when ischemic heart disease, cerebrovascular disease, and other cardiovascular disease components were all observed for a county. The other 1153 (36.8%) did not contribute to the strict composite: 1151 had one or more cardiovascular components missing, consistent with suppression of small counts, and 2 Alaska successor areas (Chugach and Copper River) had no mortality data because their deaths are carried at the legacy Valdez-Cordova code. Under the partial lower-bound sensitivity composite, which retained counties for which at least one of the three components was observed, 2765 counties (88.2%) contributed. Sample characteristics are shown in Table 1; counties outside the strict sample were smaller, older, and more often nonmetropolitan (Supplemental Table S3). Connecticut contributed no observations to the county-level analyses, for the reasons described in Section 2.9.

**Table 1:** Characteristics of counties in the strict and partial analytic samples.

Characteristic	Strict sample	Mean (SD)	Partial sample	Mean (SD)
	Median (IQR) or n (%)		Median (IQR) or n (%)	
Counties, n^a^	1982		2765	
**Mortality (2021–2023)**				
AHRF-defined cardiovascular mortality composite, crude rate per 100,000^b^^,^^c^^,^^d^	347.4 (274.5–421.2)	351.6 (104.4)	336.3 (264.8–411.2)	340.1 (107.3)
Average annual composite deaths	177 (105–387)	443 (988)	115 (52–270)	327 (856)
**Population**				
Population, 2023, thousands	50.2 (27.6–130.1)	161.9 (407.6)	31.7 (15.1–83.0)	119.3 (351.7)
**Socioeconomic measures, %**				
Pediatric poverty (ages 0–17)	17.7 (12.9–22.8)	18.3 (7.4)	18.0 (13.0–23.5)	19.0 (8.0)
Family childhood poverty (ages 5–17)	16.9 (12.1–22.0)	17.6 (7.4)	17.2 (12.4–22.7)	18.2 (8.0)
Overall poverty	13.6 (10.5–17.1)	14.1 (5.0)	13.7 (10.5–17.6)	14.5 (5.5)
Overall unemployment	4.7 (3.7–5.8)	4.9 (1.8)	4.6 (3.5–5.8)	4.9 (2.1)
White unemployment^e^	4.1 (3.2–5.1)	4.3 (1.6)	3.9 (3.0–5.1)	4.2 (1.9)
Female unemployment	4.5 (3.4–5.8)	4.8 (1.9)	4.4 (3.1–5.8)	4.7 (2.4)
**Covariates**^f^**, %**				
Aged ≥65 years	19.5 (17.1–22.1)	20.0 (4.6)	20.2 (17.6–22.9)	20.5 (4.6)
Non-Hispanic Black	4.2 (1.3–13.6)	10.6 (14.0)	3.0 (1.0–12.0)	9.9 (14.6)
Hispanic	5.8 (3.1–11.9)	10.4 (12.8)	5.2 (2.8–11.0)	10.0 (13.2)
American Indian/Alaska Native	0.6 (0.4–1.2)	1.6 (4.3)	0.7 (0.4–1.4)	2.0 (5.8)
Uninsured, age <65 years	8.8 (6.7–12.6)	9.9 (4.3)	9.1 (6.8–12.9)	10.2 (4.4)
**Rural–Urban Continuum Code 2023, n (%)**				
Metropolitan (codes 1–3)	1021 (51.5)		1154 (41.7)	
Nonmetropolitan, adjacent to metro (4, 6, 8)	671 (33.9)		963 (34.8)	
Nonmetropolitan, not adjacent (5, 7, 9)	290 (14.6)		648 (23.4)	

AHRF = HRSA Area Health Resources File (2024–2025 release); NCHS = National Center for Health Statistics; ICD-10 = International Classification of Diseases, 10th Revision; SAIPE = U.S. Census Bureau Small Area Income and Poverty Estimates; ACS = American Community Survey. IQR = interquartile range; SD = standard deviation; SAHIE = Small Area Health Insurance Estimates.

a Connecticut was excluded from county-level analyses because, following the state's 2022 transition from legacy counties to Planning Regions, no Connecticut row contains both a mortality numerator and the population and predictor data required to calculate matched county-level outcomes. Connecticut was retained in state- and Census-division-level analyses via aggregation of mortality across the 8 legacy counties and population across the 9 Planning Regions.

b Described throughout as an “AHRF-defined cardiovascular mortality composite” because HRSA does not formally label the sum of these three NCHS categories as a unified construct, and ICD-10 circulatory codes I80–I89 and I95–I99 are not included in these categories.

c Component categories: ischemic heart disease (ICD-10 I20–I25); cerebrovascular disease (I60–I69); and other cardiovascular disease (I00–I09, I10–I13, I15, I26–I51, I70, I71–I78), as listed in the AHRF 2024–2025 User Guide (Table 1). AHRF suppresses 3-year averages based on fewer than 10 occurrences.

d All reported rates are crude. AHRF does not provide age-adjusted cause-specific cardiovascular mortality measures.

e ACS White unemployment is constructed from ACS Table C23002A and includes both Hispanic and non-Hispanic White persons.

f From the AHRF 2024–2025 release: 2023 Census population estimates by age, race, and Hispanic origin (American Indian/Alaska Native is race alone and includes Hispanic persons) and 2022 SAHIE estimates for residents younger than 65 years. Values are median (IQR) and mean (SD) unless stated otherwise; definitions and sources of all variables are given in Supplemental Table S2.

### 3.2 Geographic variation in cardiovascular mortality

County-level crude cardiovascular mortality rates differed significantly across the nine U.S. Census divisions (Kruskal-Wallis H = 271.5, df = 8, p = 4.7 × 10^−54^). On a population-weighted aggregated basis, the East South Central division had the highest crude rate at 348.5 deaths per 100,000 residents, and the Mountain division had the lowest at 233.9 deaths per 100,000. The full division ranking is presented in Fig. 2.

At the state level, the highest crude cardiovascular mortality rates were observed in West Virginia (392.8 per 100,000), Mississippi (385.7), and Alabama (377.2). The lowest crude rates were observed in Utah (160.7 per 100,000), Alaska (176.0), and Colorado (188.0). State-level results are reported descriptively; no inferential omnibus test was performed at the state level. Under the strict composite, fewer than half of county-equivalents contributed in 12 states (Alaska, Colorado, Idaho, Iowa, Kansas, Montana, Nebraska, Nevada, North Dakota, South Dakota, Utah, and Wyoming), including all three lowest-rate states, so their state rates are based on a minority of their counties. The full 51-state ranking under both the strict primary and partial lower-bound composites is provided in Supplemental Table S1.

### 3.3 Socioeconomic correlates of cardiovascular mortality

All six socioeconomic predictors were positively correlated with the strict primary AHRF-defined cardiovascular mortality composite, and all correlations were statistically significant (p < 0.001). Pediatric poverty (ages 0–17) had the numerically largest coefficient (ρ = 0.612; 95% CI 0.581–0.641), followed by family childhood poverty (ages 5–17; ρ = 0.603) and overall poverty (ρ = 0.524). Among the three unemployment measures, overall unemployment had the largest coefficient (ρ = 0.209), followed by White unemployment (ρ = 0.176) and female unemployment (ρ = 0.141). The smallest poverty coefficient (overall poverty, ρ = 0.524) was larger than the largest unemployment coefficient (overall unemployment, ρ = 0.209). Coefficients with 95% CIs are shown in Table 2 and the pediatric poverty relationship in Fig. 1; all 18 correlation tests remained significant after Benjamini–Hochberg adjustment (Supplemental Table S4). The pediatric poverty coefficient exceeded the overall poverty coefficient by 0.088 (95% CI 0.071–0.107), and the overall poverty coefficient exceeded the overall unemployment coefficient by 0.315 (95% CI 0.276–0.356; both Holm-adjusted p < 0.001; Supplemental Table S5). Pediatric and family childhood poverty differed by only 0.009 (95% CI 0.005–0.013); this difference was statistically distinguishable because the measures are nearly collinear (ρ = 0.993 between them) but is negligible in magnitude.

**Table 2:** **Unadjusted and adjusted county-level associations between socioeconomic measures and the AHRF-defined cardiovascular mortality composite** Strict complete-component composite (main outcome); n = 1982 counties.

Socioeconomic measure	Unadjusted Spearman ρ (95% CI)	Model A: state fixed effects (95% CI)	Model B: main adjusted model (95% CI)	Model C: Model B + insurance (95% CI)
Poverty				
Pediatric poverty (ages 0–17)	0.612 (0.581–0.641)	0.580 (0.546–0.612)	0.530 (0.495–0.563)	0.515 (0.479–0.554)
Family childhood poverty (ages 5–17)	0.603 (0.572–0.633)	0.573 (0.539–0.606)	0.522 (0.487–0.555)	0.508 (0.471–0.546)
Overall poverty	0.524 (0.489–0.558)	0.463 (0.422–0.502)	0.461 (0.421–0.499)	0.445 (0.404–0.490)
**Unemployment**				
Overall unemployment	0.209 (0.165–0.252)	0.198 (0.154–0.243)	0.209 (0.163–0.255)	0.204 (0.157–0.251)
White unemployment^a^	0.176 (0.131–0.220)	0.204 (0.159–0.248)	0.189 (0.141–0.234)	0.186 (0.137–0.232)
Female unemployment	0.141 (0.098–0.186)	0.105 (0.061–0.150)	0.165 (0.118–0.212)	0.160 (0.113–0.208)
**Joint model (overall poverty and overall unemployment)**^b^				
Overall poverty, adjusted for overall unemployment	—	0.429 (0.385–0.469)	0.421 (0.378–0.461)	0.405 (0.361–0.452)
Overall unemployment, adjusted for overall poverty	—	−0.039 (−0.086 to 0.008)	0.029 (−0.020 to 0.077)	0.031 (−0.018 to 0.079)

Values are Spearman ρ (unadjusted) or partial rank correlations (Models A–C; Pearson correlation of county rank residuals after ordinary least-squares adjustment) with 95% county-level bias-corrected and accelerated bootstrap confidence intervals (10,000 resamples). All adjusted models include state fixed effects. Model A: state fixed effects only. Model B (main adjusted model): Model A plus the percentage of residents aged ≥65 years, the 2023 Rural–Urban Continuum Code (nine categories), and the percentages of non-Hispanic Black, Hispanic, and American Indian/Alaska Native residents. Model C: Model B plus the percentage of residents younger than 65 years without health insurance (2022 SAHIE). Adjustment for the percentage aged ≥65 years is covariate adjustment for age structure; rates are crude, not age-adjusted. Connecticut was excluded from county-level analyses. Sensitivity analyses, the complementary population-weighted negative binomial model, and the contiguity-based spatial sensitivity analysis are in Supplemental Table S6–S8.

a ACS White unemployment includes both Hispanic and non-Hispanic White persons.

b Joint model containing both overall poverty and overall unemployment; each row gives the partial association of one measure adjusted for the other and for the model covariates.

**Fig. 1:**
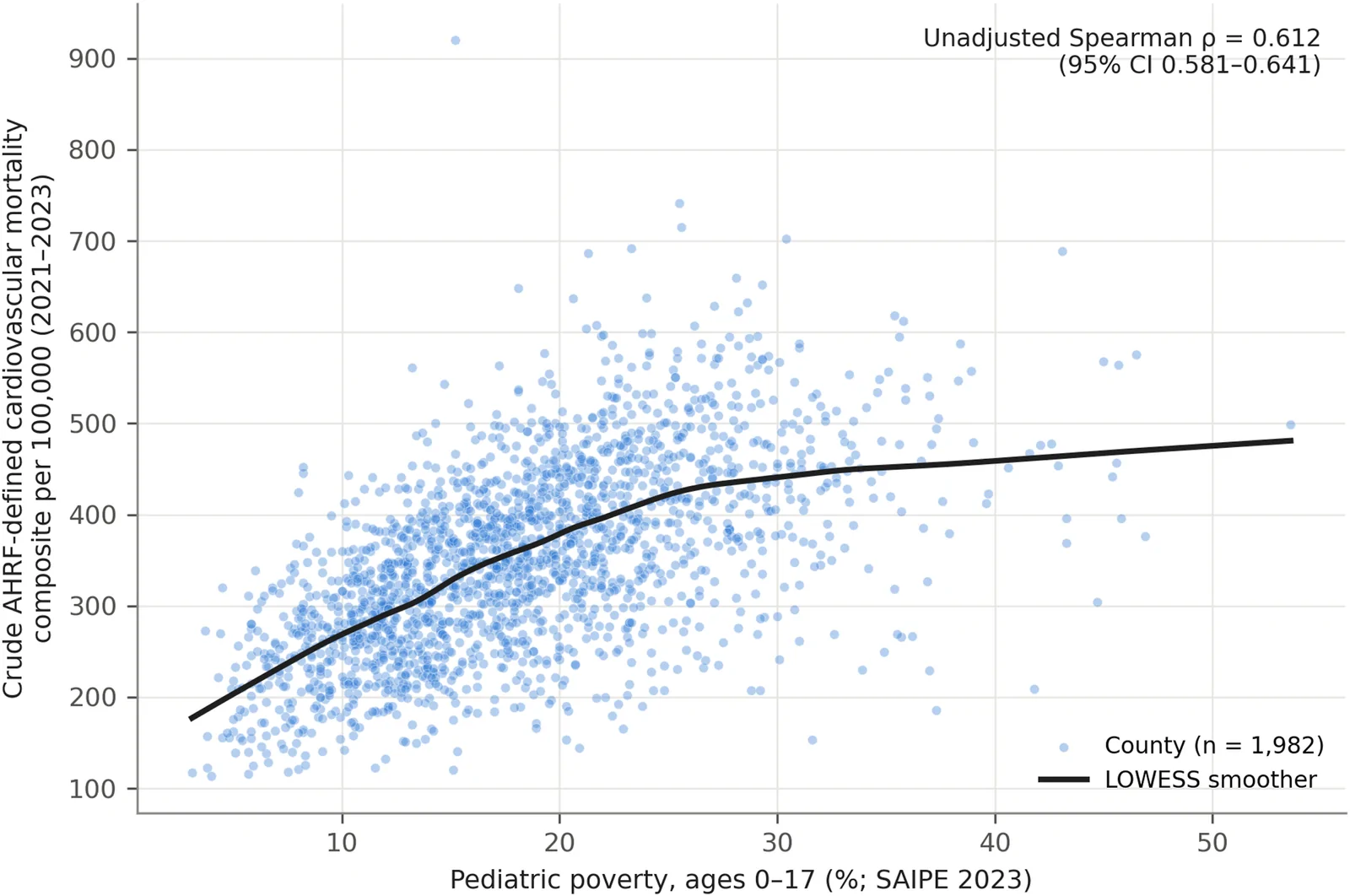
**Pediatric Poverty and the AHRF-Defined Cardiovascular Mortality Composite Across U.S. Counties** County-level pediatric poverty (percent of persons aged 0–17 in poverty, 2023 SAIPE) versus the crude AHRF-defined cardiovascular mortality composite (3-year average annual deaths per 100,000 residents, 2021–2023; strict complete-component composite) for 1982 counties in the 50 states and the District of Columbia; Connecticut is excluded from county-level analyses. Each point is one county; the line is a LOWESS smoother (span 0.3). Unadjusted Spearman ρ = 0.612 (95% CI 0.581–0.641; county-level BCa bootstrap); adjusted estimates are given in Table 2. Unadjusted correlations for all six socioeconomic measures are shown in Supplemental Fig. S1. Mortality rates are crude (not age-adjusted). • The cardiovascular mortality outcome is an AHRF-defined composite. It is not labeled by HRSA as “total cardiovascular mortality”; it sums the three non-overlapping NCHS cardiovascular cause-of-death categories carried in AHRF, which do not include ICD-10 circulatory codes I80–I89 or I95–I99. • Rates are crude, not age-adjusted. AHRF does not publish age-adjusted cause-specific rates. • A partial lower-bound sensitivity composite (which includes counties with one or two missing NCHS components, consistent with suppression) preserved the rank order of predictors, with smaller poverty and larger unemployment coefficients (Supplemental Table S4); the figures shown use the strict primary outcome.

• Source: HRSA Area Health Resources File (AHRF) 2024–2025 release; NCHS Mortality Detail Data Files; U.S. Census Bureau (County Population Estimates and SAIPE); American Community Survey 2019–2023 5-year tables.

**Fig. 2:**
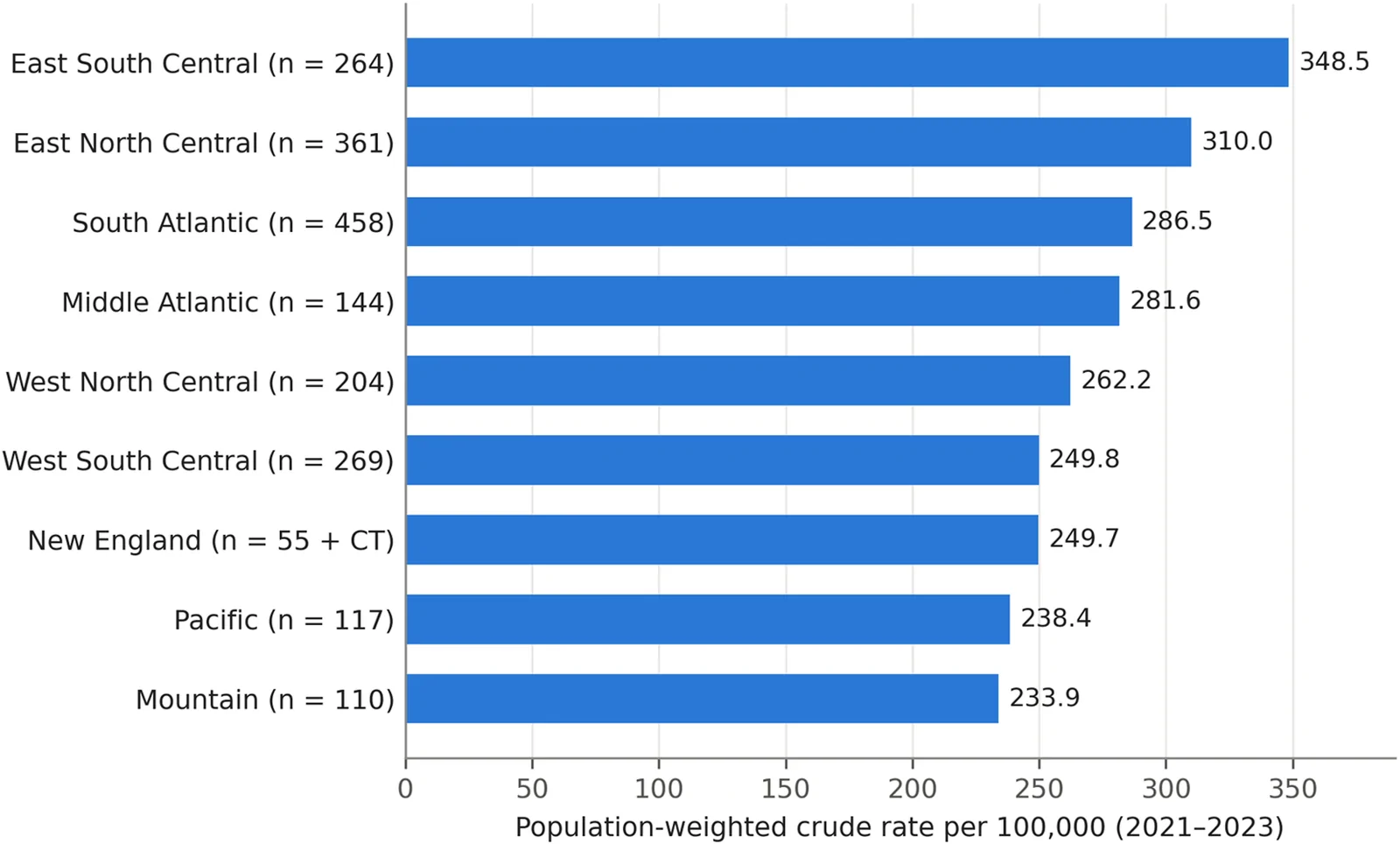
**Cardiovascular Mortality Rates by U.S. Census Division** Population-weighted aggregated crude cardiovascular mortality rates per 100,000 residents for each of the nine U.S. Census divisions, 2021–2023. Rates use the strict complete AHRF-defined cardiovascular mortality composite (ischemic heart + cerebrovascular + other cardiovascular disease) and 2023 Census County Population Estimates. Divisions are ordered from highest (East South Central, 348.5 per 100,000) to lowest (Mountain, 233.9 per 100,000). Labels give the number of contributing counties in each division. Connecticut is included in the New England division through a state-level aggregation that combines the 8 legacy-county mortality numerator with the 9 Planning Region population denominator; this aggregation reflects the geographic boundary of the state and is consistent with Connecticut's inclusion at the state level. County-level Kruskal-Wallis test of regional variation: H = 271.5, df = 8, p = 4.7 × 10^−54^ (Connecticut excluded from county-level testing). Mortality rates are crude (not age-adjusted). • The cardiovascular mortality outcome is an AHRF-defined composite. It is not labeled by HRSA as “total cardiovascular mortality”; it sums the three non-overlapping NCHS cardiovascular cause-of-death categories carried in AHRF, which do not include ICD-10 circulatory codes I80–I89 or I95–I99. • Rates are crude, not age-adjusted. AHRF does not publish age-adjusted cause-specific rates. • A partial lower-bound sensitivity composite (which includes counties with one or two missing NCHS components, consistent with suppression) preserved the rank order of predictors, with smaller poverty and larger unemployment coefficients (Supplemental Table S4); the figures shown use the strict primary outcome.

• Source: HRSA Area Health Resources File (AHRF) 2024–2025 release; NCHS Mortality Detail Data Files; U.S. Census Bureau (County Population Estimates and SAIPE); American Community Survey 2019–2023 5-year tables.

### 3.4 Adjusted analyses

In the main adjusted model (Model B; Table 2), partial rank correlations were 0.530 (95% CI 0.495–0.563) for pediatric poverty, 0.522 for family childhood poverty, and 0.461 for overall poverty, versus 0.165–0.209 for unemployment measures. In the joint model, overall poverty remained associated with the outcome after adjustment for overall unemployment (0.421; 95% CI 0.378–0.461), whereas overall unemployment adjusted for overall poverty was close to zero (0.029; 95% CI −0.020 to 0.077). Between-state differences accounted for 24% of county variance in the composite rate and 34% in pediatric poverty (Supplemental Table S9). Estimates were similar with Census-division fixed effects, three-category rurality, and Spearman correlation of residuals (Supplemental Table S6). In negative binomial models, a 1-SD (7.4 percentage-point) higher pediatric poverty corresponded to a rate ratio of 1.17 (95% CI 1.15–1.19), versus 1.03–1.05 for unemployment measures (Supplemental Table S7). Allowing correlation between contiguous counties increased standard errors (up to 1.42-fold unadjusted, 1.11-fold adjusted) without changing conclusions (Supplemental Table S8).

### 3.5 Sensitivity analyses

Under the partial lower-bound composite (n = 2765 counties), coefficients were smaller than under the strict composite for all three poverty measures (pediatric poverty, 0.612 to 0.535) and larger for all three unemployment measures (overall unemployment, 0.209 to 0.226), with unchanged rank order (Supplemental Table S4). Because this outcome both adds 783 counties and uses a lower-bound numerator for them, these shifts cannot be attributed to either change alone. East South Central and Mountain remained the highest and lowest divisions. The three highest- and lowest-rate states were unchanged. Under Kendall τ as an alternative nonparametric correlation metric, the rank order of the six predictors against the strict primary composite was again identical, with pediatric poverty the largest and female unemployment the smallest coefficient.

## 4 Discussion

### 4.1 Principal findings

In this national county-level analysis, the AHRF-defined cardiovascular mortality composite showed marked geographic variation across U.S. counties, with the highest population-weighted aggregated crude rate in the East South Central Census division and the lowest in the Mountain division. Among six county-level socioeconomic measures, the three poverty measures showed numerically larger associations with cardiovascular mortality than the three unemployment measures, and pediatric poverty (ages 0–17) had the numerically largest coefficient. After adjustment for county age structure, rurality, racial and ethnic composition, and state, poverty measures retained larger partial associations than unemployment measures, and the association of overall unemployment was near zero once overall poverty was accounted for. The interpretation that follows is ecological and is presented as a population-level pattern rather than a statement about individual-level causation.

### 4.2 Geographic variation in context

The geographic pattern observed here is consistent with the persistent concentration of high cardiovascular mortality in the South and Appalachia, and lower mortality in the Mountain West, described in national county-level analyses [2], [3], [4], [5]. Our results add a contemporary 2021–2023 view using an AHRF-defined cardiovascular mortality composite; rankings are not directly comparable with earlier work because outcome construction, period, denominators, and aggregation differ.

### 4.3 Socioeconomic deprivation and cardiovascular mortality

Recent state-of-the-art reviews of the social determinants of cardiovascular health identify economic stability, including income and material resources, as an important social determinant of cardiovascular health [10]. Our finding that poverty measures showed larger associations with the AHRF-defined cardiovascular mortality composite than unemployment measures, both unadjusted and after covariate adjustment, is consistent with this broader framing.

### 4.4 Interpreting the pediatric poverty association

That pediatric poverty (ages 0–17) had the numerically largest coefficient among the six socioeconomic measures examined invites careful interpretation in light of the existing life-course literature. Its margin over family childhood poverty, a nearly collinear measure, was negligible. Individual-level studies link childhood adversity and socioeconomic disadvantage to later cardiometabolic risk through stress-related, biological, and behavioral pathways [11], [12], [13] and are consistent with a life-course association between childhood disadvantage and adult cardiovascular risk [14], [15]. Importantly, our ecological analysis cannot determine whether current pediatric poverty prevalence directly caused later cardiovascular mortality among any specific individuals; the children represented in 2023 SAIPE poverty estimates are not the same individuals whose deaths contribute to the 2021–2023 mortality counts. We therefore interpret the association between county-level pediatric poverty and the AHRF-defined cardiovascular mortality composite as a county-level signal of broader, sustained socioeconomic disadvantage, including likely intergenerational patterns of deprivation, rather than as evidence of an individual-level causal chain from a child's poverty to that child's later cardiovascular death.

### 4.5 Poverty versus unemployment as county-level measures

The weaker associations observed for unemployment measures than for poverty measures with the AHRF-defined cardiovascular mortality composite should not be interpreted as suggesting that unemployment is unimportant for cardiovascular health. At the county level, however, unemployment rates may function as a narrower and more temporally variable marker of economic conditions than poverty, and may not fully capture underemployment, labor-force nonparticipation, retirement, disability, household resource availability, food insecurity, housing instability, or intergenerational disadvantage. Poverty measures may therefore approximate sustained county-level deprivation more closely than unemployment in cross-sectional analyses of cardiovascular mortality.

### 4.6 Public health implications

These findings suggest that readily available county-level poverty measures, including pediatric poverty, are candidate markers of county cardiovascular mortality burden, measured here by the AHRF-defined cardiovascular mortality composite, that warrant further investigation, including longitudinal analyses [6], [11]. The findings are cross-sectional and based on crude rates, do not evaluate any policy or intervention, and should be interpreted alongside the substantial body of work emphasizing that improvements in cardiovascular health are unlikely to be achieved through health-care interventions alone. Cross-sector strategies such as Health in All Policies and the related Health in All Laws proposal [17] address social conditions upstream of health care; the present study does not evaluate their effectiveness.

### 4.7 Limitations

This study has several limitations. First, the design is ecological and cross-sectional; county-level associations cannot establish individual-level causation, and pediatric poverty in this analysis reflects current 2023 county-level prevalence rather than the childhood circumstances of decedents. Second, all rates are crude. AHRF provides no age-specific cause-specific deaths, and county-level age-adjusted rates for this period are not available from CDC WONDER [18]. Adjustment for the percentage aged ≥65 years addresses age structure only as a covariate. This percentage was strongly correlated with the crude rate (ρ = 0.586) but weakly with pediatric poverty (ρ = 0.134), and the pediatric poverty association persisted after adjustment; residual confounding by age structure, of uncertain direction, remains possible, and state and division differences may partly reflect age structure. Third, 1153 counties (36.8%) did not contribute to the strict primary composite: 1151 because one or more cardiovascular components were missing, consistent with suppression of small counts, so inclusion depends partly on the outcome, and 2 Alaska areas because their deaths are recorded under a legacy code. The partial composite retained 783 of these counties but with a lower-bound numerator, and its coefficients shifted in opposite directions for poverty and unemployment measures. Excluded counties were smaller, older, and more rural (Supplemental Table S3), so the findings apply mainly to relatively larger, more metropolitan counties. Fourth, Connecticut's 2022 county-to-Planning-Region transition required exclusion from county-level analyses and use of a documented state- and division-level aggregation. Fifth, the analytic variables are derived from different temporal windows: 2021–2023 mortality, 2023 SAIPE single-year poverty, and 2019–2023 ACS 5-year unemployment, introducing modest temporal mismatch. Sixth, ecological analyses of this kind remain susceptible to residual confounding, including by county smoking and obesity prevalence, which AHRF does not provide, and to within-county heterogeneity, and findings should not be generalized to individual-level associations. Seventh, White unemployment was a labor-market measure, not a control for racial and ethnic composition, which the adjusted models included. Eighth, count-model residuals were spatially autocorrelated (Supplemental Table S7), and spatial dependence was addressed only through a contiguity-based sensitivity analysis.

## 5 Conclusion

In this contemporary national county-level analysis, poverty measures, including pediatric poverty, showed larger associations with the AHRF-defined cardiovascular mortality composite than unemployment measures, both unadjusted and after covariate adjustment. County pediatric poverty is a candidate marker of county cardiovascular mortality burden that warrants further investigation; this ecological finding, based on crude rates, is not evidence of individual-level causation.

## CRediT authorship contribution statement

**Kevin Babapour Digaleh:** Writing – original draft, Methodology, Formal analysis, Data curation. **Mahdi Bouchekouk:** Formal analysis, Data curation. **Ronen Bina:** Formal analysis, Data curation. **Wyatt House:** Writing – original draft. **Toshihito Gomibuchi:** Writing – original draft. **Alyster Alcudia:** Writing – original draft. **Medha Tekriwal:** Writing – review & editing, Writing – original draft. **Suyog Mokashi:** Writing – review & editing, Supervision.

## Disclosures

The authors have no financial relationships or conflicts of interest relevant to this work to disclose.

## Ethics statement

Attached.

## Data availability

The 2024–2025 Area Health Resources File used in this analysis is publicly available from the U.S. Health Resources and Services Administration at https://data.hrsa.gov/topics/health-workforce/ahrf. Underlying county-level cause-of-death counts are available from the CDC WONDER platform (https://wonder.cdc.gov). Analytic code and derived intermediate datasets supporting the results reported here are available from the corresponding author upon reasonable request.

## Author agreement form

This statement is to certify that all authors have seen and approved the manuscript being submitted, have contributed significantly to the work, attest to the validity and legitimacy of the data and its interpretation, and agree to its submission to the *International Journal of Cardiology Cardiovascular Risk and Prevention*.

We attest that the article is the Authors' original work, has not received prior publication and is not under consideration for publication elsewhere. On behalf of all Co-Authors, the corresponding Author shall bear full responsibility for the submission. Any changes to the list of authors, including changes in order, additions or removals will require the submission of a new author agreement form approved and signed by all the original and added submitting authors.

## Declaration of generative AI and AI-assisted technologies in the manuscript preparation process

No AI was used in the manuscript preparation process.

## Funding

No funding was received for this work.

## Declaration of competing interest

The authors declare that they have no known competing financial interests or personal relationships that could have appeared to influence the work reported in this paper.
