# Supplementary material for: Pediatric poverty and county-level cardiovascular mortality in the United States: A national cross-sectional analysis
Source: Int J Cardiol Cardiovasc Risk Prev. 2026 Oct 30;31:200722. doi: 10.1016/j.ijcrp.2026.200722 (PMC13760060; doi:10.1016/j.ijcrp.2026.200722)
Supplement: Supplementary file 1 [file mmc1.pdf]

# Supplemental Material

Pediatric Poverty and County-Level Cardiovascular Mortality in the United States: A National Cross-Sectional Analysis

*Manuscript IJCCRP-D-26-00556 (revision). Draft for author review, 7 October 2026.*

### Contents


- Supplemental Methods (sections M1–M11)
- Supplemental Table S1. State-level rates and county coverage (provided as a separate file)
- Supplemental Table S2. Variable definitions and data sources
- Supplemental Table S3. Characteristics of counties included in and excluded from the strict analytic sample
- Supplemental Table S4. Unadjusted county-level correlations with 95% CIs and false discovery rate–adjusted p-values
- Supplemental Table S5. Paired contrasts between unadjusted Spearman correlations
- Supplemental Table S6. Partial rank correlations: sensitivity analyses
- Supplemental Table S7. Negative binomial rate ratios and model diagnostics
- Supplemental Table S8. Contiguity-based spatial sensitivity analysis
- Supplemental Table S9. Within- and between-state variation and fixed- versus random-effects comparison
- Supplemental Figure S1. Unadjusted Spearman correlations for six socioeconomic measures

# Supplemental Methods

## M1. Timing and provenance of the analyses added during peer review

The submitted manuscript reported unadjusted Spearman and Kendall correlations, a Kruskal–Wallis test across Census divisions, and descriptive state and division summaries. The confidence intervals, paired contrasts, false discovery rate adjustment, adjusted analyses, count models, and spatial and other sensitivity analyses described below were added in response to peer review. The study statistician selected these methods on 7 October 2026, after provisional outputs of candidate analyses (28 September 2026) had been produced and shared with him. The methods were therefore not prespecified or preregistered. Implementation details that the statistician did not specify were fixed in a dated written specification before the final outputs were computed, and every specified analysis is reported regardless of its result. Provisional analyses that were not selected (Fisher-z and Bonett–Wright intervals, a state-cluster bootstrap, a percentile-interval decision rule for the contrasts, a quasi-Poisson rate model, and an imputation-based upper-value scenario for missing components) are not reported. Before any reported value was replaced, the originally reported correlations, Kruskal–Wallis statistic, and state and division summaries were reproduced from the analytic file, and key new estimates were recomputed with independent code.

## M2. County accounting and missing data

The AHRF 2024–2025 release contains 3,153 county records for the 50 states and the District of Columbia. Of these, 17 are Connecticut records (8 legacy counties and 9 Planning Regions) and 1 is the dissolved Valdez-Cordova Census Area, Alaska (FIPS 02261), leaving 3,135 county-equivalents. 1,982 contributed to the strict composite (all three components observed). Of the other 1,153, 1,151 had one or more components missing and 2 (Chugach, 02063, and Copper River, 02066) have population and predictor data but no mortality data because their deaths are carried at the legacy code. The partial lower-bound composite added 783 counties (589 with one and 194 with two components missing), for 2,765; 368 counties had no component observed. AHRF suppresses 3-year averages based on fewer than 10 occurrences and carries suppressed and unreported values identically as missing, so missing components are described as consistent with suppression rather than as documented suppression. No missing component was imputed. Every count above was re-derived from the analytic file and matched the manuscript.

## M3. Covariates

All covariates came from the same AHRF release (Supplemental Table S2): the percentage of residents aged ≥65 years and the percentages of non-Hispanic Black, Hispanic, and American Indian/Alaska Native residents (2023 Census population estimates; American Indian/Alaska Native is race alone and includes Hispanic persons); the 2023 Rural–Urban Continuum Code (RUCC), entered as a nine-level factor with code 1 as reference; and, in Model C only, the percentage of residents younger than 65 years without health insurance (2022 Small Area Health Insurance Estimates). Every covariate was complete in both analytic samples. Adjustment for the percentage aged ≥65 years is covariate adjustment for county age structure; it does not convert the outcome into an age-adjusted rate, and no direct or indirect standardization was performed. Across the 1,982 strict-sample counties, the percentage aged ≥65 years was correlated with the crude composite rate (Spearman $\rho = 0.586$) and only weakly with pediatric poverty ($\rho = 0.134$). County smoking and obesity prevalence were not available: a search of all column names in the eight AHRF county files, the Technical Documentation, and the User Guide found no

smoking, tobacco-use, obesity, or body-mass-index prevalence measure. External model-based estimates were not added (see manuscript Limitations).

## M4. Confidence intervals for Spearman correlations

Ninety-five percent confidence intervals for every reported Spearman ρ were obtained by county-level bias-corrected and accelerated (BCa) bootstrap: 10,000 resamples of counties with replacement (seed 20260928), with the outcome and all six socioeconomic measures resampled together, variables re-ranked within every resample (average ranks for ties), and the acceleration constant estimated by the jackknife. The strict-composite, partial-composite, and added-county correlations each used their own resamples. Intervals were flagged as unstable if the adjusted quantile levels fell outside 0.001–0.999; none were flagged. Kendall τ is reported as a coefficient with its p-value only.

## M5. Paired contrasts between correlations

Three contrasts chosen by the statistician were estimated as differences of strict Spearman ρ computed on the same 10,000 county resamples: (a) pediatric minus family childhood poverty; (b) pediatric minus overall poverty; and (c) overall poverty minus overall unemployment. Each difference is reported with a 95% BCa interval for estimation. Distinguishability was judged by a centered two-sided bootstrap p-value, $p = (1 + \#\{|d^*_b - \hat{d}| \geq |\hat{d}|\})/(B + 1)$, with Holm's step-down adjustment across the three contrasts (adjusted $p < 0.05$). The smallest attainable p-value is $1/(B + 1) \approx 0.0001$; all three contrasts were at this floor and are reported as $p < 0.001$. As validity checks, the p-values were recomputed from the saved draws with independent code (they matched); the bootstrap distributions were nearly symmetric (skewness 0.04 to 0.06); and normal-approximation p-values were all well below 0.001 (Supplemental Table S5). Steiger's test was not used because it is derived for Pearson correlations under bivariate normality; the bootstrap accounts for the shared outcome and the dependence between measures directly. Contrast (a) compares two nearly collinear measures (Spearman ρ = 0.993 between them), so a small difference is statistically distinguishable; it is described by its magnitude, and distinguishability is not interpreted as meaningful superiority.

## M6. Multiplicity

The Benjamini–Hochberg false discovery rate procedure was applied to the 18 correlation tests counted by the reviewer: six socioeconomic measures × (strict Spearman ρ, partial-composite Spearman ρ, strict Kendall τ), the 18 cells of the original Table 2. The largest adjusted p-value was $3.7\times10^{-10}$. Kendall τ for the partial composite was computed in the original analysis but never reported; those six tests (which would make 24) are outside the family and are not reported. The Kruskal–Wallis test and the three paired contrasts are separate families.

## M7. Adjusted analysis: partial rank correlations

The main adjusted analysis estimated an equal-weight county partial rank correlation. The outcome and the socioeconomic measure were ranked across the strict sample; each set of ranks was regressed by ordinary least squares on the model covariates and state fixed effects; and the estimate is the Pearson correlation of the two residual vectors. Model A included state fixed effects only, Model B (main) added the covariates in M3 except insurance, and Model C added insurance; the change from Model B to Model C is interpreted as attenuation conditional on insurance, not as mediation. The joint model contained overall poverty and overall unemployment together (residualized on each other and the covariates by the Frisch–Waugh–Lovell construction). No model contained more than one poverty measure. A model containing all six measures was not fitted because the measures are highly collinear:

in the strict sample, pediatric poverty had a variance inflation factor of 61 and family childhood poverty 53 relative to the other five measures. Intervals are 95% county BCa bootstrap intervals (10,000 resamples, seed 20260928), re-ranking and refitting within every resample; no design was rank-deficient. Sensitivity analyses replaced state with Census-division fixed effects (Models A–C), coded RUCC in three categories (metropolitan 1–3; nonmetropolitan adjacent 4, 6, 8; nonmetropolitan nonadjacent 5, 7, 9), and used the Spearman rather than the Pearson correlation of the residuals (Supplemental Table S6).

## M8. Negative binomial models

As a complementary, population-weighted analysis, NB2 negative binomial regression (maximum likelihood, log link, dispersion $\alpha$ estimated) modeled the strict 2021–2023 average annual composite deaths as published by AHRF, with log(2023 population) as offset. Each socioeconomic measure was standardized by its unweighted standard deviation in the strict sample, so rate ratios are per 1-SD difference. Specifications were Model 0 (no covariates), Models A–C with state fixed effects (Model B is the main complementary specification), Models A–C with Census-division fixed effects, Model B with three-category RUCC, and the joint model. Standard errors were cluster-robust by state (CR1); no state random intercept was included alongside state fixed effects. All models converged. In Model B with state fixed effects, $\alpha$ ranged from 0.018 to 0.025, Pearson $\chi^2$/df from 1.00 to 1.02, and observed/expected deaths by decile of fitted deaths from 0.94 to 1.12; 9–12 counties had |Pearson residual| > 3, and the Akaike information criterion favored the negative binomial over the Poisson model in every specification. Because the negative binomial likelihood is not scale-invariant, Model B was refitted to approximate 3-year totals (deaths × 3, offset log(3 × population)); the pediatric poverty rate ratio changed from 1.170 to 1.168. Moran's I of the Model B Pearson residuals (row-standardized first-order contiguity, 999 permutations) was 0.14–0.18 (permutation p = 0.001 for each), indicating residual spatial autocorrelation (M9). The rate ratios describe crude, population-weighted death rates and answer a different question from the equal-weight partial rank correlations; they are not presented as validating them (Supplemental Table S7).

## M9. Contiguity-based spatial sensitivity analysis

County adjacency was taken from the AHRF geography file (first-order contiguous counties), symmetrized and restricted to the strict sample: 4,631 neighbor pairs, median 5 neighbors per county, and 32 counties with no in-sample neighbor, which contribute only their own term. No external data were used and no spatial lag model was fitted. Standard errors allowing correlation between each county and its contiguous neighbors were computed with a Conley-type spatial heteroskedasticity- and autocorrelation-consistent estimator with a uniform kernel (weight 1 for a county with itself and with each first-order neighbor, 0 otherwise), applied to the influence function of the correlation of standardized ranks, $\psi = z_x z_y - (\rho/2)(z_x^2 + z_y^2)$, for the unadjusted correlations and contrasts, to the standardized rank residuals for the main partial rank correlations, and to the score contributions (sandwich estimator) for the main negative binomial model. Ranks and covariate projections were treated as fixed. The same estimator under independence is reported alongside as a check against the bootstrap; for the unadjusted correlations it reproduced the bootstrap standard errors. Contiguity increased standard errors by factors of up to 1.42 for the unadjusted correlations and up to 1.11 for the partial rank correlations; all three contrasts remained distinguishable after Holm adjustment, and the joint-model unemployment interval still included zero (Supplemental Table S8). The written specification stated the influence function in slope form, $\psi = z_x(z_y - \rho z_x)$; the correlation form above was implemented in code fixed before any output was computed and is reported. As a check, recomputing with the slope form changed the contiguity standard errors by factors of 1.01–1.12

(unadjusted), 1.00–1.16 (partial rank), and 1.05–1.19 (contrasts), without changing any conclusion. Under independence, the correlation form reproduced the bootstrap standard errors of the unadjusted correlations (ratio 0.99–1.00), whereas the slope form exceeded them for the poverty measures (ratio up to 1.10).

## M10. Within- and between-state variation

Between-state differences accounted for 24% of the county variance (one-way sum-of-squares decomposition) in the crude composite rate and 34% in pediatric poverty across the 50 jurisdictions with strict-sample counties (median 31 counties per jurisdiction, range 1–134). Because the adjusted analyses use state fixed effects, the partial correlations reflect within-state variation. A Hausman-type comparison of state fixed-effects and state random-intercept slopes (rank framework, Model B covariates) is reported in Supplemental Table S9 as indicative only, because its conventional variances assume independent errors, which the contiguity diagnostics contradict; state fixed effects were the statistician's choice for the main analysis, with Census-division fixed effects as a sensitivity analysis.

## M11. Software and reproducibility

Analyses were run in Python 3.11.15 with numpy 2.4.4, pandas 3.0.2, scipy 1.17.1, and statsmodels 0.15.0. Each output records the code version, the specification checksum, and the run time. Key results were recomputed by independent code paths: the partial rank correlations by direct regression, BCa intervals with an independent bootstrap implementation, negative binomial coefficients and cluster-robust standard errors by separate routines, and the false discovery rate, Holm, contiguity, and descriptive calculations; all agreed within numerical tolerance. Analysis code and derived datasets are available from the corresponding author on reasonable request.

## Supplemental Table S1

Supplemental Table S1 (state-level population-weighted crude rates and county and population coverage under the strict and partial composites, 50 states and the District of Columbia) is provided as a separate file.

## Supplemental Table S2. Variable definitions and data sources

| Measure | Source and period | Definition or analytic sample |
| --- | --- | --- |
| **Analytic sample** | | |
| Geographic universe | AHRF 2024–2025; U.S. Census Bureau geography | 50 states and the District of Columbia: 3,153 AHRF county records; U.S. territories excluded |
| Connecticut | AHRF 2024–2025 | Excluded from county-level analyses (mortality reported for legacy counties, population and predictors for Planning Regions); included in state and division summaries by state-level aggregation |
| Strict analytic sample | Counties with all three composite components observed | n = 1,982 counties |
| Partial analytic sample | Counties with at least one component observed | n = 2,765 counties |
| **Outcomes** | | |
| Strict AHRF-defined cardiovascular mortality composite (main outcome) | NCHS mortality data in AHRF; 2021–2023 3-year average annual deaths | Sum of ischemic heart disease (ICD-10 I20–I25), cerebrovascular disease (I60–I69), and other cardiovascular disease (I00–I09, I10–I13, I15, I26–I51, I70, I71–I78) deaths; defined only when all three components are observed. Crude rate per 100,000 with the 2023 Census population estimate as denominator |
| Partial lower-bound composite (sensitivity outcome) | As above | Sum of observed components when at least one is observed; a lower bound where one or two components are missing (missing values consistent with suppression of small counts). Same denominator |
| **Socioeconomic measures** | | |
| Overall poverty | SAIPE; 2023 single-year estimate | Percent of all persons in poverty |
| Pediatric poverty (ages 0–17) | SAIPE; 2023 single-year estimate | Percent of persons aged 0–17 in poverty |
| Family childhood poverty (ages 5–17) | SAIPE; 2023 single-year estimate | Percent of related children aged 5–17 in families in poverty |
| Overall unemployment | ACS 2019–2023 5-year (Table B23001) | Unemployed persons aged ≥16 as a percent of the civilian labor force aged ≥16 |
| Female unemployment | ACS 2019–2023 5-year (Table B23001) | Unemployed females aged ≥16 as a percent of the female civilian labor force aged ≥16 |
| White unemployment | ACS 2019–2023 5-year (Table C23002A) | Unemployed White persons aged ≥16 as a percent of the White civilian labor force aged ≥16; includes Hispanic and non-Hispanic White persons |
| **Covariates (adjusted analyses)** | | |
| Aged ≥65 years | Census 2023 population estimates in AHRF | Population aged ≥65 as a percent of total population (covariate adjustment for age structure; not age standardization) |
| Non-Hispanic Black | Census 2023 population estimates in AHRF | Non-Hispanic Black population as a percent of total population |
| Hispanic | Census 2023 population estimates in AHRF | Hispanic population (any race) as a percent of total population |

| Measure | Source and period | Definition or analytic sample |
|---|---|---|
| American Indian/Alaska Native | Census 2023 population estimates in AHRF | American Indian/Alaska Native population (race alone, including Hispanic persons) as a percent of total population |
| Rural–Urban Continuum Code | USDA ERS 2023 codes in AHRF | Nine categories (codes 1–9; reference code 1); three-category version in sensitivity analyses: metropolitan (1–3), nonmetropolitan adjacent to a metropolitan area (4, 6, 8), nonmetropolitan not adjacent (5, 7, 9) |
| Uninsured, age <65 years (Model C only) | SAHIE 2022 in AHRF | Percent of persons younger than 65 without health insurance |
| State; Census division | AHRF geography | Fixed effects for state (main) or Census division (sensitivity) |

ACS = American Community Survey; AHRF = Area Health Resources File; ERS = Economic Research Service; NCHS = National Center for Health Statistics; SAHIE = Small Area Health Insurance Estimates; SAIPE = Small Area Income and Poverty Estimates; USDA = U.S. Department of Agriculture. ICD-10 circulatory codes I80–I89 and I95–I99 are not included in the composite. AHRF suppresses 3-year averages based on fewer than 10 occurrences and carries suppressed and unreported values identically as missing. All rates are crude.

## Supplemental Table S3. Characteristics of counties included in and excluded from the strict analytic sample

| Characteristic | Strict sample (n = 1,982) | Added by the partial composite (n = 783) | No component observed (n = 368) | SMD, added vs strict | SMD, none vs strict |
|---|---|---|---|---|---|
| Population, 2023, persons | 50,188 (27,610–130,130) | 10,438 (7,683–14,352) | 2,987 (1,866–4,763) | −0.52 | −0.55 |
| Population aged ≥65, % | 19.5 (17.1–22.1) | 21.6 (19.3–24.2) | 24.1 (19.5–27.8) | 0.43 | 0.65 |
| Non-Hispanic Black, % | 4.2 (1.3–13.6) | 1.2 (0.7–5.2) | 0.9 (0.5–1.7) | −0.16 | −0.67 |
| Hispanic, % | 5.8 (3.1–11.9) | 3.9 (2.4–8.1) | 5.7 (3.0–17.1) | −0.10 | 0.24 |
| AIAN, race alone, % | 0.6 (0.4–1.2) | 0.9 (0.5–1.8) | 1.5 (1.0–3.0) | 0.20 | 0.40 |
| Uninsured, age <65, % | 8.8 (6.7–12.6) | 9.7 (7.2–13.7) | 11.7 (8.4–16.2) | 0.18 | 0.56 |
| Pediatric poverty (ages 0–17), % | 17.7 (12.9–22.8) | 18.9 (13.6–26.1) | 17.1 (13.3–22.6) | 0.28 | 0.10 |
| Family childhood poverty (ages 5–17), % | 16.9 (12.1–22.0) | 18.0 (12.8–25.0) | 16.4 (12.9–22.1) | 0.28 | 0.14 |
| Overall poverty, % | 13.6 (10.5–17.1) | 14.1 (10.8–19.0) | 12.6 (10.7–16.1) | 0.26 | 0.02 |
| Overall unemployment, % | 4.7 (3.7–5.8) | 4.1 (2.8–5.9) | 2.5 (1.2–4.5) | −0.10 | −0.42 |
| White unemployment, % | 4.1 (3.2–5.1) | 3.5 (2.3–5.3) | 1.8 (0.9–3.7) | −0.14 | −0.85 |
| Female unemployment, % | 4.5 (3.4–5.8) | 3.7 (2.2–5.9) | 2.0 (0.6–4.4) | −0.11 | −0.48 |
| Metropolitan (RUCC 1–3), % | 51.5 | 17.0 | 6.8 | — | — |
| Nonmetro adjacent (RUCC 4/6/8), % | 33.9 | 37.3 | 22.0 | — | — |
| Nonmetro nonadjacent (RUCC 5/7/9), % | 14.6 | 45.7 | 71.2 | — | — |
| Most rural (RUCC 8–9), % | 11.6 | 63.1 | 89.4 | — | — |

Values are median (interquartile range) or percent. SMD = standardized mean difference, (mean of the comparison group − mean of the strict sample)/pooled SD, on the raw scale; |SMD| ≥ 0.1 is commonly regarded as a meaningful imbalance. Counties were not contributing to the strict sample mainly because components were missing (consistent with suppression of small counts), so inclusion depends partly on the outcome. The two structurally missing Alaska areas are excluded from all three columns and are shown individually: Chugach (02063): population 6,769, aged ≥65 18.0%, RUCC 9, pediatric poverty 7.9%; Copper River (02066): population 2,674, aged ≥65 20.5%, RUCC 9, pediatric poverty 20.1%. Uninsured and SAIPE values are missing for one county with no component observed. No statistical tests were performed.

## Supplemental Table S4. Unadjusted county-level correlations with 95% CIs and false discovery rate–adjusted p-values

| Socioeconomic measure | Strict composite (n = 1,982) Spearman ρ (95% CI) | BH-adjusted p | Partial composite (n = 2,765) Spearman ρ (95% CI) | BH-adjusted p | Strict composite Kendall τ | BH-adjusted p | Added counties (n = 783) Spearman ρ (95% CI) |
|---|---|---|---|---|---|---|---|
| Pediatric poverty (ages 0–17) | 0.612 (0.581–0.641) | $3.7\times10^{-203}$ | 0.535 (0.505–0.563) | $3.7\times10^{-203}$ | 0.437 | $7.3\times10^{-186}$ | 0.442 (0.377–0.501) |
| Family childhood poverty (ages 5–17) | 0.603 (0.572–0.633) | $4.8\times10^{-196}$ | 0.526 (0.497–0.555) | $4.8\times10^{-196}$ | 0.431 | $1.3\times10^{-180}$ | 0.435 (0.370–0.494) |
| Overall poverty | 0.524 (0.489–0.558) | $7.6\times10^{-140}$ | 0.461 (0.428–0.491) | $7.9\times10^{-145}$ | 0.370 | $8.1\times10^{-134}$ | 0.386 (0.317–0.447) |
| Overall unemployment | 0.209 (0.165–0.252) | $8.1\times10^{-21}$ | 0.226 (0.190–0.262) | $3.4\times10^{-33}$ | 0.141 | $8.1\times10^{-21}$ | 0.220 (0.148–0.286) |
| White unemployment | 0.176 (0.131–0.220) | $2.9\times10^{-15}$ | 0.196 (0.159–0.234) | $3.1\times10^{-25}$ | 0.120 | $1.6\times10^{-15}$ | 0.191 (0.121–0.259) |
| Female unemployment | 0.141 (0.098–0.186) | $3.1\times10^{-10}$ | 0.166 (0.128–0.203) | $2.1\times10^{-18}$ | 0.094 | $3.7\times10^{-10}$ | 0.160 (0.088–0.228) |

CIs are 95% county-level bias-corrected and accelerated bootstrap intervals (10,000 resamples). BH-adjusted p-values use the Benjamini–Hochberg procedure across the 18 tests in the first six numeric columns (6 measures × strict ρ, partial ρ, strict Kendall τ). The added-county correlations use the partial (lower-bound) rate in the 783 counties that enter only under the partial composite; they are descriptive and do not separate the effect of adding counties from that of the lower-bound numerator. BH = Benjamini–Hochberg.

## Supplemental Table S5. Paired contrasts between unadjusted Spearman correlations (strict composite, n = 1,982)

| Contrast | ρ, first measure | ρ, second measure | Difference (95% CI) | Bootstrap SE | Bootstrap skewness | Centered bootstrap p | Holm-adjusted p | Normal-approximation p (check) | Spearman ρ between the two measures |
|---|---|---|---|---|---|---|---|---|---|
| (a) Pediatric poverty − family childhood poverty | 0.612 | 0.603 | 0.009 (0.005–0.013) | 0.0022 | 0.04 | <0.001 | <0.001 | $4.9\times10^{-5}$ | 0.993 |
| (b) Pediatric poverty − overall poverty | 0.612 | 0.524 | 0.088 (0.071–0.107) | 0.0090 | 0.06 | <0.001 | <0.001 | $1.6\times10^{-22}$ | 0.938 |
| (c) Overall poverty − overall unemployment | 0.524 | 0.209 | 0.315 (0.276–0.356) | 0.0202 | 0.05 | <0.001 | <0.001 | $4.2\times10^{-55}$ | 0.535 |

Differences and 95% bias-corrected and accelerated intervals were computed on the same 10,000 county resamples. Decision rule: centered two-sided bootstrap $p = (1 + \#\{|d^*_b - \hat{d}| \geq |\hat{d}|\})/(B + 1)$, Holm-adjusted across the three contrasts; the smallest attainable p is 1/10,001 ≈ 0.0001, and values at this floor are shown as <0.001. The normal-approximation p (difference/bootstrap SE) is a sanity check only. Statistical distinguishability is not interpreted as meaningful superiority; contrast (a) compares nearly collinear measures and is negligible in magnitude.

## Supplemental Table S6. Partial rank correlations: sensitivity analyses (strict composite, n = 1,982)

| Socioeconomic measure | Model B, state FE (main; Table 2) | Model A, division FE | Model B, division FE | Model C, division FE | Model B, state FE, 3-category RUCC | Model B, state FE, Spearman of residuals |
|---|---|---|---|---|---|---|
| Pediatric poverty (ages 0–17) | 0.530 (0.495–0.563) | 0.587 (0.554–0.617) | 0.542 (0.510–0.574) | 0.535 (0.503–0.567) | 0.559 (0.527–0.590) | 0.524 (0.489–0.561) |
| Family childhood poverty (ages 5–17) | 0.522 (0.487–0.555) | 0.577 (0.543–0.609) | 0.536 (0.503–0.567) | 0.528 (0.495–0.560) | 0.553 (0.521–0.584) | 0.517 (0.482–0.554) |
| Overall poverty | 0.461 (0.421–0.499) | 0.490 (0.451–0.527) | 0.483 (0.447–0.519) | 0.479 (0.443–0.515) | 0.497 (0.459–0.533) | 0.456 (0.418–0.497) |
| Overall unemployment | 0.209 (0.163–0.255) | 0.232 (0.189–0.275) | 0.240 (0.196–0.283) | 0.248 (0.204–0.290) | 0.221 (0.175–0.266) | 0.208 (0.163–0.254) |
| White unemployment | 0.189 (0.141–0.234) | 0.240 (0.196–0.281) | 0.232 (0.187–0.274) | 0.239 (0.195–0.281) | 0.200 (0.152–0.244) | 0.191 (0.144–0.237) |
| Female unemployment | 0.165 (0.118–0.212) | 0.144 (0.099–0.188) | 0.206 (0.161–0.252) | 0.208 (0.163–0.254) | 0.170 (0.122–0.217) | 0.173 (0.128–0.220) |
| Overall poverty (joint model) | 0.421 (0.378–0.461) | 0.445 (0.405–0.484) | 0.432 (0.393–0.472) | 0.424 (0.384–0.465) | 0.457 (0.416–0.495) | — |

| Socioeconomic measure | Model B, state FE (main; Table 2) | Model A, division FE | Model B, division FE | Model C, division FE | Model B, state FE, 3-category RUCC | Model B, state FE, Spearman of residuals |
|---|---|---|---|---|---|---|
| Overall unemployment (joint model) | 0.029 (−0.020 to 0.077) | −0.039 (−0.085 to 0.007) | 0.023 (−0.025 to 0.070) | 0.032 (−0.016 to 0.079) | 0.024 (−0.025 to 0.073) | — |

Values are partial rank correlations (Pearson correlation of OLS rank residuals unless stated) with 95% county-level bias-corrected and accelerated bootstrap intervals (10,000 resamples, re-ranking and refitting in each resample). Model A: geographic fixed effects only; Model B: Model A plus percentage aged ≥65 years, Rural–Urban Continuum Code (nine categories unless stated), and percentages non-Hispanic Black, Hispanic, and American Indian/Alaska Native; Model C: Model B plus percentage younger than 65 years uninsured. Joint model: overall poverty and overall unemployment together, each adjusted for the other. The Spearman-of-residuals variant was not computed for the joint model (—). FE = fixed effects.

## Supplemental Table S7. Negative binomial rate ratios and model diagnostics (strict composite, n = 1,982)

### A. Rate ratio per 1-SD higher value of each measure (95% CI)

| Socioeconomic measure | 1 SD (points) | Model 0 (no covariates) | Model A, state FE | Model B, state FE (main) | Model C, state FE | Model A, division FE | Model B, division FE | Model C, division FE | Model B, state FE, 3-category RUCC |
|---|---|---|---|---|---|---|---|---|---|
| Pediatric poverty (ages 0–17) | 7.4 | 1.199 (1.172–1.227) | 1.201 (1.178–1.226) | 1.170 (1.153–1.189) | 1.165 (1.149–1.182) | 1.201 (1.177–1.226) | 1.172 (1.149–1.194) | 1.169 (1.147–1.190) | 1.178 (1.160–1.196) |
| Family childhood poverty (ages 5–17) | 7.4 | 1.195 (1.167–1.224) | 1.199 (1.175–1.224) | 1.171 (1.154–1.189) | 1.166 (1.149–1.182) | 1.198 (1.172–1.224) | 1.173 (1.150–1.196) | 1.170 (1.147–1.192) | 1.179 (1.161–1.198) |
| Overall poverty | 5.0 | 1.166 (1.139–1.193) | 1.150 (1.126–1.174) | 1.133 (1.115–1.151) | 1.126 (1.109–1.143) | 1.159 (1.136–1.182) | 1.138 (1.115–1.160) | 1.136 (1.116–1.155) | 1.141 (1.124–1.158) |
| Overall unemployment | 1.8 | 1.068 (1.039–1.098) | 1.059 (1.041–1.078) | 1.047 (1.032–1.061) | 1.044 (1.028–1.060) | 1.072 (1.053–1.091) | 1.054 (1.040–1.069) | 1.057 (1.042–1.072) | 1.050 (1.034–1.066) |
| White unemployment | 1.6 | 1.055 (1.023–1.088) | 1.059 (1.038–1.080) | 1.037 (1.024–1.050) | 1.036 (1.022–1.049) | 1.071 (1.050–1.092) | 1.047 (1.034–1.060) | 1.050 (1.037–1.063) | 1.041 (1.026–1.055) |
| Female unemployment | 1.9 | 1.048 (1.018–1.079) | 1.034 (1.017–1.052) | 1.033 (1.020–1.046) | 1.031 (1.017–1.045) | 1.048 (1.027–1.068) | 1.044 (1.029–1.059) | 1.045 (1.030–1.060) | 1.035 (1.022–1.049) |
| Overall poverty (joint model) | 5.0 | 1.182 (1.152–1.213) | 1.158 (1.131–1.185) | 1.130 (1.111–1.149) | 1.122 (1.105–1.140) | 1.164 (1.138–1.190) | 1.134 (1.109–1.159) | 1.130 (1.107–1.152) | 1.139 (1.120–1.159) |
| Overall unemployment (joint model) | 1.8 | 0.976 (0.951–1.001) | 0.988 (0.970–1.006) | 1.005 (0.992–1.018) | 1.006 (0.993–1.019) | 0.993 (0.974–1.012) | 1.006 (0.992–1.020) | 1.009 (0.996–1.023) | 1.003 (0.990–1.017) |

### B. Diagnostics, Model B with state fixed effects

| Socioeconomic measure | Dispersion α (95% CI) | Pearson $\chi^2$/df | Counties with \|Pearson residual\| > 3 | AIC, negative binomial / Poisson | Observed/expected by decile of fitted deaths | Model-based/robust SE ratio | Moran's I of residuals | Rate ratio, annual / 3-year scale |
|---|---|---|---|---|---|---|---|---|
| Pediatric poverty (ages 0–17) | 0.018 (0.016–0.019) | 1.01 | 11 | 19,753 / 32,433 | 0.96–1.08 | 0.77 | 0.14 | 1.170 / 1.168 |
| Family childhood poverty (ages 5–17) | 0.018 (0.017–0.019) | 1.01 | 12 | 19,775 / 32,797 | 0.96–1.08 | 0.79 | 0.14 | 1.171 / 1.169 |
| Overall poverty | 0.020 (0.018–0.021) | 1.00 | 9 | 19,934 / 33,399 | 0.95–1.09 | 0.73 | 0.14 | 1.133 / 1.131 |
| Overall unemployment | 0.024 (0.023–0.026) | 1.02 | 11 | 20,267 / 36,180 | 0.94–1.12 | 0.71 | 0.17 | 1.047 / 1.044 |
| White unemployment | 0.025 (0.023–0.027) | 1.02 | 11 | 20,285 / 36,322 | 0.94–1.12 | 0.76 | 0.17 | 1.037 / 1.035 |
| Female unemployment | 0.025 (0.023–0.027) | 1.02 | 9 | 20,302 / 36,780 | 0.94–1.12 | 0.80 | 0.18 | 1.033 / 1.030 |

NB2 negative binomial regression of 2021–2023 average annual composite deaths (as published by AHRF) with log(2023 population) offset; cluster-robust (CR1) standard errors by state; measures standardized by their unweighted SD in the strict sample. Models are as in Supplemental Table S6; Model 0 has no covariates or fixed effects. All models converged. Moran's I: row-standardized first-order contiguity, 999 permutations, p = 0.001 for each measure. Rate ratio, 3-year scale: Model B refitted with deaths × 3 and offset log(3 × population), because the negative binomial likelihood is not scale-invariant. Rate ratios describe crude, population-weighted rates. AIC = Akaike information criterion; FE = fixed effects.

## Supplemental Table S8. Contiguity-based spatial sensitivity analysis (strict composite, n = 1,982)

| Estimate | Value | SE, independence | SE, contiguity | SE ratio | 95% CI, contiguity SE | Holm-adjusted p, contiguity SE |
|---|---|---|---|---|---|---|
| **Unadjusted Spearman ρ** | | | | | | |
| Pediatric poverty (ages 0–17) | 0.612 | 0.0152 | 0.0214 | 1.41 | 0.570–0.654 | |
| Family childhood poverty (ages 5–17) | 0.603 | 0.0154 | 0.0218 | 1.41 | 0.560–0.646 | |
| Overall poverty | 0.524 | 0.0176 | 0.0243 | 1.38 | 0.476–0.572 | |
| Overall unemployment | 0.209 | 0.0218 | 0.0303 | 1.39 | 0.149–0.268 | |
| White unemployment | 0.176 | 0.0224 | 0.0318 | 1.42 | 0.114–0.239 | |
| Female unemployment | 0.141 | 0.0221 | 0.0291 | 1.32 | 0.084–0.198 | |
| **Paired contrasts (difference in ρ)** | | | | | | |
| (a) Pediatric poverty (ages 0–17) minus family childhood poverty (ages 5–17) | 0.009 | 0.0022 | 0.0023 | 1.05 | 0.004–0.013 | <0.001 |
| (b) Pediatric poverty (ages 0–17) minus overall poverty | 0.088 | 0.0089 | 0.0106 | 1.18 | 0.067–0.109 | <0.001 |
| (c) Overall poverty minus overall unemployment | 0.315 | 0.0201 | 0.0272 | 1.35 | 0.262–0.368 | <0.001 |
| **Partial rank correlation, Model B, state fixed effects (main adjusted analysis)** | | | | | | |
| Pediatric poverty (ages 0–17) | 0.530 | 0.0169 | 0.0187 | 1.10 | 0.493–0.566 | |
| Family childhood poverty (ages 5–17) | 0.522 | 0.0169 | 0.0187 | 1.11 | 0.485–0.559 | |
| Overall poverty | 0.461 | 0.0195 | 0.0211 | 1.08 | 0.420–0.502 | |
| Overall unemployment | 0.209 | 0.0233 | 0.0252 | 1.08 | 0.160–0.258 | |
| White unemployment | 0.189 | 0.0236 | 0.0261 | 1.10 | 0.138–0.240 | |
| Female unemployment | 0.165 | 0.0237 | 0.0240 | 1.02 | 0.117–0.212 | |
| Overall poverty (joint model) | 0.421 | 0.0207 | 0.0220 | 1.07 | 0.378–0.464 | |
| Overall unemployment (joint model) | 0.029 | 0.0244 | 0.0252 | 1.03 | −0.020 to 0.079 | |
| **Negative binomial rate ratio per 1 SD, Model B, state fixed effects** | | | | | | |
| Pediatric poverty (ages 0–17) | 1.170 | 0.0069 | 0.0081 | 1.18 | 1.152–1.189 | |
| Family childhood poverty (ages 5–17) | 1.171 | 0.0074 | 0.0086 | 1.17 | 1.152–1.191 | |
| Overall poverty | 1.133 | 0.0071 | 0.0083 | 1.16 | 1.115–1.152 | |

| Estimate | Value | SE, independence | SE, contiguity | SE ratio | 95% CI, contiguity SE | Holm-adjusted p, contiguity SE |
|---|---|---|---|---|---|---|
| Overall unemployment | 1.047 | 0.0052 | 0.0058 | 1.11 | 1.035–1.058 | |
| White unemployment | 1.037 | 0.0048 | 0.0054 | 1.11 | 1.026–1.048 | |
| Female unemployment | 1.033 | 0.0052 | 0.0055 | 1.05 | 1.022–1.044 | |

Conley-type spatial heteroskedasticity- and autocorrelation-consistent standard errors with a uniform kernel over each county and its first-order contiguous neighbors (AHRF geography file; 4,631 neighbor pairs; 32 counties without an in-sample neighbor). Independence SEs use the same estimator with weights only on the diagonal. Wald intervals; ranks and covariate projections treated as fixed; negative binomial SEs and intervals are on the log scale and back-transformed. With the slope-form influence function stated in the written specification, contiguity SEs were 1.00–1.19 times those shown, with no change in conclusions (Supplemental Methods M9). Sensitivity analysis only; SE = standard error.

## Supplemental Table S9. Within- and between-state variation and fixed- versus random-effects comparison

### A. Variance decomposition by state (strict sample)

| Variable | Between-state share of variance, % | Within-state share of variance, % | Intraclass correlation (state random intercept) |
|---|---|---|---|
| Crude composite rate | 23.7 | 76.3 | 0.28 |
| Pediatric poverty (ages 0–17) | 34.2 | 65.8 | 0.38 |

### B. Rank slope of the outcome on each measure, Model B covariates: state fixed effects vs state random intercept

| Socioeconomic measure | Fixed effects, slope (SE) | Random intercept, slope (SE) | Hausman $\chi^2$ (1 df) | p |
|---|---|---|---|---|
| Pediatric poverty (ages 0–17) | 0.509 (0.019) | 0.520 (0.018) | 12.89 | 0.0003 |
| Family childhood poverty (ages 5–17) | 0.509 (0.019) | 0.521 (0.019) | 12.13 | 0.0005 |
| Overall poverty | 0.413 (0.018) | 0.422 (0.018) | 11.66 | 0.0006 |
| Overall unemployment | 0.155 (0.017) | 0.156 (0.016) | 0.01 | 0.9158 |
| White unemployment | 0.129 (0.015) | 0.130 (0.015) | 0.20 | 0.6580 |
| Female unemployment | 0.118 (0.016) | 0.120 (0.016) | 1.07 | 0.3014 |

Panel A: one-way sum-of-squares decomposition across the 50 jurisdictions with strict-sample counties, and the intraclass correlation from a null linear mixed model (REML). Panel B: ordinary least-squares slope of the outcome rank on the measure rank with Model B covariates and state fixed effects, versus the same model with a state random intercept (REML). The Hausman-type statistic assumes independent errors, which the contiguity diagnostics contradict, so it is indicative only. SE = standard error.

## Supplemental Figure S1. Unadjusted Spearman correlations for six socioeconomic measures

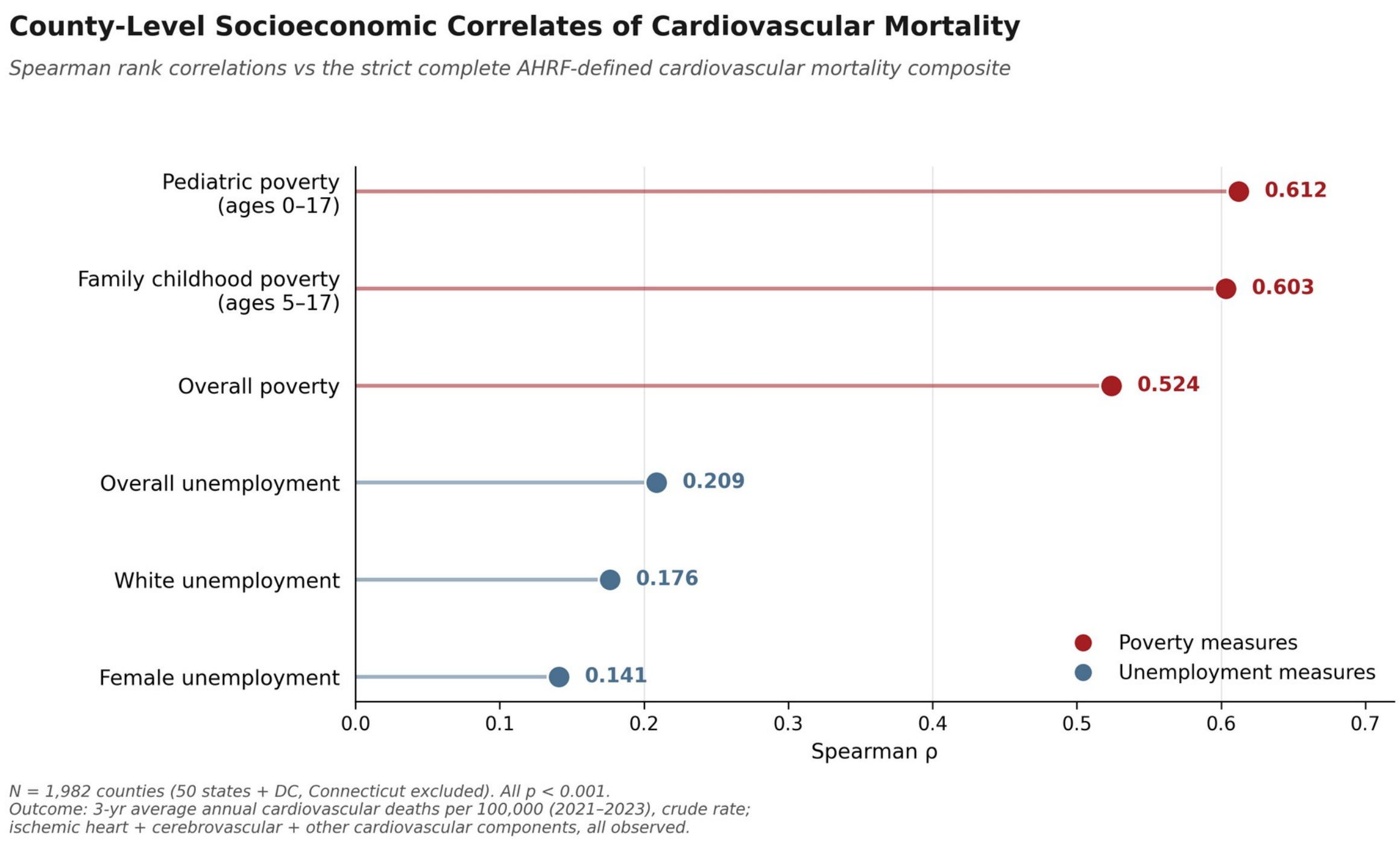


Spearman rank correlations between six county socioeconomic measures and the strict AHRF-defined cardiovascular mortality composite (crude 2021–2023 rate; n = 1,982 counties; Connecticut excluded). This is Figure 1 of the submitted manuscript, unchanged and moved to the supplement during revision; 95% confidence intervals are given in Supplemental Table S4 and adjusted estimates in Table 2 of the manuscript.
