# Supplementary material for: Pediatric poverty and county-level cardiovascular mortality in the United States: A national cross-sectional analysis
Source: Int J Cardiol Cardiovasc Risk Prev. 2026 Oct 30;31:200722. doi: 10.1016/j.ijcrp.2026.200722 (PMC13760060; doi:10.1016/j.ijcrp.2026.200722)
Supplement: Supplementary file 2 [file mmc2.pdf]

Supplemental Table S1. State-level population-weighted crude rates of the AHRF-defined cardiovascular mortality composite, 2021–2023, ranked separately under the strict (primary) and partial lower-bound (sensitivity) composites, 50 states and the District of Columbia

DRAFT for author review, rebuilt 2026-09-26 from the verified prepared county file; the originally submitted S1 has not been obtained.

Panel A. Strict complete-component composite (primary) [b] — ranked by strict-composite rate

Rank	State	Census division	County-equivalents, n [c]	Contributing, n (%) [e]	Population coverage, % [e]	Average annual deaths [a]	Population [a]	Crude rate per 100,000 [a]
1	West Virginia	South Atlantic	55	37 (67.3)	90.6	6,297	1,602,956	392.8
2	Mississippi	East South Central	82	60 (73.2)	93.1	10,553	2,736,251	385.7
3	Alabama	East South Central	67	58 (86.6)	98.1	18,902	5,011,309	377.2
4	Oklahoma	West South Central	77	41 (53.2)	91.5	13,150	3,710,619	354.4
5	Arkansas	West South Central	75	48 (64.0)	90.7	9,748	2,781,419	350.5
6	Michigan	East North Central	83	71 (85.5)	98.9	34,287	9,928,215	345.3
7	Louisiana	West South Central	64	46 (71.9)	95.1	14,614	4,351,744	335.8
8	Ohio	East North Central	88	84 (95.5)	99.5	39,251	11,731,838	334.6
9	Tennessee	East South Central	95	80 (84.2)	97.9	23,167	6,973,320	332.2
10	Maine	New England	16	16 (100.0)	100.0	4,459	1,395,722	319.5
11	Pennsylvania	Middle Atlantic	67	63 (94.0)	99.8	41,153	12,930,552	318.3
12	Florida	South Atlantic	67	61 (91.0)	99.7	71,065	22,540,399	315.3
13	Kentucky	East South Central	120	66 (55.0)	86.0	12,253	3,894,609	314.6
14	Missouri	West North Central	115	67 (58.3)	92.4	17,910	5,724,209	312.9
15	Delaware	South Atlantic	3	3 (100.0)	100.0	3,227	1,031,890	312.7
16	South Carolina	South Atlantic	46	43 (93.5)	99.4	16,151	5,343,271	302.3
17	Vermont	New England	14	11 (78.6)	93.9	1,835	607,927	301.8
18	Indiana	East North Central	92	75 (81.5)	96.7	19,319	6,638,652	291.0
19	Nevada	Mountain	17	8 (47.1)	98.3	9,092	3,140,575	289.5
20	Rhode Island	New England	5	5 (100.0)	100.0	3,111	1,095,962	283.9
21	Wisconsin	East North Central	72	54 (75.0)	95.7	16,027	5,658,162	283.3
22	Iowa	West North Central	99	32 (32.3)	74.4	6,748	2,385,227	282.9
23	Illinois	East North Central	102	77 (75.5)	98.3	34,607	12,333,663	280.6
24	Hawaii	Pacific	5	4 (80.0)	100.0	3,946	1,435,057	275.0
25	Kansas	West North Central	105	24 (22.9)	78.5	6,306	2,307,268	273.3
26	North Carolina	South Atlantic	100	89 (89.0)	98.9	29,063	10,713,582	271.3
27	New York	Middle Atlantic	62	60 (96.8)	99.9	52,958	19,548,627	270.9
28	New Mexico	Mountain	33	21 (63.6)	95.4	5,451	2,016,962	270.3
29	Oregon	Pacific	36	28 (77.8)	98.9	11,273	4,185,021	269.4
30	New Hampshire	New England	10	10 (100.0)	100.0	3,776	1,402,054	269.3
31	Maryland	South Atlantic	24	24 (100.0)	100.0	16,471	6,180,253	266.5
32	Montana	Mountain	56	10 (17.9)	73.4	2,203	832,022	264.8
33	Arizona	Mountain	15	14 (93.3)	99.9	19,599	7,421,975	264.1
34	Virginia	South Atlantic	133	99 (74.4)	95.6	21,199	8,329,296	254.5
35	Georgia	South Atlantic	159	101 (63.5)	95.0	26,624	10,477,048	254.1
36	New Jersey	Middle Atlantic	21	21 (100.0)	100.0	23,528	9,290,841	253.2
37	Wyoming	Mountain	23	7 (30.4)	63.8	937	372,741	251.4
38	Connecticut [d]	New England	9	Aggregate [d]	100.0	8,890	3,617,176	245.8
39	California	Pacific	58	51 (87.9)	99.8	92,356	38,882,448	237.5
40	Nebraska	West North Central	93	16 (17.2)	78.0	3,634	1,542,971	235.5
41	Dist. of Columbia	South Atlantic	1	1 (100.0)	100.0	1,597	678,972	235.2
42	South Dakota	West North Central	66	10 (15.2)	65.4	1,366	601,201	227.2
43	Massachusetts	New England	14	13 (92.9)	99.8	15,647	6,986,955	223.9
44	Washington	Pacific	39	30 (76.9)	98.8	17,248	7,716,077	223.5
45	Idaho	Mountain	44	17 (38.6)	86.0	3,724	1,689,503	220.4
46	Texas	West South Central	254	134 (52.8)	97.3	63,710	29,673,701	214.7
47	Minnesota	West North Central	87	48 (55.2)	92.3	11,163	5,293,447	210.9
48	North Dakota	West North Central	53	7 (13.2)	67.8	1,086	531,814	204.2
49	Colorado	Mountain	64	22 (34.4)	92.5	10,222	5,438,256	188.0
50	Alaska [c]	Pacific	30	4 (13.3)	76.0	981	557,377	176.0
51	Utah	Mountain	29	11 (37.9)	92.8	5,096	3,171,332	160.7

Panel B. Partial lower-bound composite (sensitivity) [b] — ranked by partial-composite rate

Rank	State	Census division	County-equivalents, n [c]	Contributing, n (%) [e]	Population coverage, % [e]	Average annual deaths [a]	Population [a]	Crude rate per 100,000 [a]
1	Mississippi	East South Central	82	81 (98.8)	100.0	11,356	2,938,434	386.5
2	West Virginia	South Atlantic	55	55 (100.0)	100.0	6,839	1,770,071	386.4
3	Alabama	East South Central	67	67 (100.0)	100.0	19,329	5,108,468	378.4
4	Oklahoma	West South Central	77	70 (90.9)	99.4	14,404	4,029,352	357.5
5	Arkansas	West South Central	75	75 (100.0)	100.0	10,926	3,067,732	356.2
6	Michigan	East North Central	83	82 (98.8)	100.0	34,729	10,035,089	346.1
7	Louisiana	West South Central	64	63 (98.4)	99.9	15,480	4,568,981	338.8
8	Ohio	East North Central	88	88 (100.0)	100.0	39,439	11,785,935	334.6
9	Tennessee	East South Central	95	95 (100.0)	100.0	23,731	7,126,489	333.0
10	Maine	New England	16	16 (100.0)	100.0	4,459	1,395,722	319.5
11	Pennsylvania	Middle Atlantic	67	67 (100.0)	100.0	41,249	12,961,683	318.2
12	Kentucky	East South Central	120	117 (97.5)	99.7	14,286	4,510,345	316.7
13	Missouri	West North Central	115	107 (93.0)	99.5	19,458	6,164,335	315.7
14	Florida	South Atlantic	67	67 (100.0)	100.0	71,276	22,610,726	315.2
15	Delaware	South Atlantic	3	3 (100.0)	100.0	3,227	1,031,890	312.7
16	South Carolina	South Atlantic	46	46 (100.0)	100.0	16,267	5,373,555	302.7
17	Vermont	New England	14	14 (100.0)	100.0	1,922	647,464	296.9
18	Iowa	West North Central	99	98 (99.0)	99.9	9,474	3,203,460	295.7
19	Indiana	East North Central	92	92 (100.0)	100.0	19,969	6,862,199	291.0
20	Nevada	Mountain	17	12 (70.6)	99.5	9,174	3,178,366	288.6
21	Rhode Island	New England	5	5 (100.0)	100.0	3,111	1,095,962	283.9
22	Wisconsin	East North Central	72	71 (98.6)	99.9	16,725	5,906,729	283.2
23	Illinois	East North Central	102	99 (97.1)	99.9	35,229	12,536,852	281.0
24	Hawaii	Pacific	5	4 (80.0)	100.0	3,946	1,435,057	275.0
25	North Carolina	South Atlantic	100	98 (98.0)	99.9	29,449	10,827,423	272.0
26	Kansas	West North Central	105	60 (57.1)	94.8	7,569	2,788,955	271.4
27	New York	Middle Atlantic	62	61 (98.4)	100.0	53,021	19,566,134	271.0
28	New Mexico	Mountain	33	30 (90.9)	99.7	5,681	2,107,967	269.5
29	Oregon	Pacific	36	33 (91.7)	99.9	11,388	4,227,945	269.4
30	New Hampshire	New England	10	10 (100.0)	100.0	3,776	1,402,054	269.3
31	Maryland	South Atlantic	24	24 (100.0)	100.0	16,471	6,180,253	266.5
32	Arizona	Mountain	15	14 (93.3)	99.9	19,599	7,421,975	264.1
33	Montana	Mountain	56	34 (60.7)	95.1	2,792	1,077,549	259.1
34	Georgia	South Atlantic	159	147 (92.5)	99.6	28,260	10,980,343	257.4
35	Virginia	South Atlantic	133	130 (97.7)	99.7	22,204	8,689,470	255.5
36	New Jersey	Middle Atlantic	21	21 (100.0)	100.0	23,528	9,290,841	253.2
37	Connecticut [d]	New England	9	Aggregate [d]	100.0	8,890	3,617,176	245.8
38	Nebraska	West North Central	93	51 (54.8)	93.7	4,510	1,853,843	243.3
39	Wyoming	Mountain	23	19 (82.6)	92.8	1,297	541,910	239.3
40	California	Pacific	58	55 (94.8)	100.0	92,521	38,947,786	237.6
41	Dist. of Columbia	South Atlantic	1	1 (100.0)	100.0	1,597	678,972	235.2
42	South Dakota	West North Central	66	32 (48.5)	88.1	1,834	810,374	226.3
43	Massachusetts	New England	14	14 (100.0)	100.0	15,660	7,001,399	223.7
44	Washington	Pacific	39	38 (97.4)	100.0	17,461	7,810,517	223.6
45	Idaho	Mountain	44	34 (77.3)	97.4	4,236	1,913,752	221.3
46	Texas	West South Central	254	189 (74.4)	99.3	65,448	30,293,585	216.0
47	North Dakota	West North Central	53	23 (43.4)	89.4	1,505	701,219	214.6
48	Minnesota	West North Central	87	80 (92.0)	99.3	12,194	5,699,380	214.0
49	Colorado	Mountain	64	40 (62.5)	97.6	10,678	5,737,070	186.1
50	Alaska [c]	Pacific	30	9 (30.0)	87.7	1,106	643,222	171.9
51	Utah	Mountain	29	24 (82.8)	99.6	5,507	3,404,594	161.8

Notes. Rank 1 = highest rate within each panel; no ties occurred. A state's strict and partial ranks can differ; the side-by-side comparison is in the accompanying spreadsheet (Supplemental_Table_S1_DRAFT.xlsx).

[a] Average annual deaths are sums, across contributing county-equivalents, of AHRF 2021–2023 three-year average annual death counts; population is their summed 2023 Census population estimate; crude rate = deaths ÷ population × 100,000, rounded to one decimal from full precision. Rates are not age-adjusted.

[b] Strict composite: ischemic heart disease + cerebrovascular disease + other cardiovascular disease (ICD-10 codes as listed in AHRF 2024–2025 User Guide Table 14), defined only when all three components are observed. Partial composite: sum of observed components when at least one is observed; a lower bound for county-equivalents with one or two missing components. AHRF suppresses three-year averages based on fewer than 10 occurrences and carries suppressed and unreported values identically as missing.

[c] County-equivalents are AHRF records with a 2023 population estimate (3,144 nationally). Alaska excludes the dissolved Valdez-Cordova Census Area record (FIPS 02261), whose 2021–2023 deaths are carried at the legacy code; its successor areas, Chugach (02063) and Copper River (02066), have population but no mortality data (structurally missing) and do not contribute under either composite.

[d] Connecticut: excluded from county-level analyses because its mortality data are reported for 8 legacy counties and its population and predictor data for 9 Planning Regions after the 2022 transition. The state rate aggregates deaths from all 8 legacy counties (all three components observed) over the population of all 9 Planning Regions; county coverage is therefore not applicable, and population coverage is 100% by construction. Connecticut is included in the New England division total by the same rule.

[e] Contributing, n (%): county-equivalents with a defined composite rate, as a percentage of county-equivalents. Population coverage: 2023 population of contributing county-equivalents as a percentage of the state 2023 population. Under the strict composite, fewer than 50% of county-equivalents contributed in 12 states.

Source: Health Resources and Services Administration, Area Health Resources Files 2024–2025 release; NCHS mortality and U.S. Census Bureau population data as carried in AHRF.
